# A Novel Ferroptosis-Related LncRNA Pair Prognostic Signature Predicts Immune Landscapes and Treatment Responses for Gastric Cancer Patients

**DOI:** 10.3389/fgene.2022.899419

**Published:** 2022-06-20

**Authors:** Jiazheng Li, Renshen Xiang, Wei Song, Jing Wu, Can Kong, Tao Fu

**Affiliations:** Department of Gastrointestinal Surgery II, Renmin Hospital of Wuhan University, Wuhan, China

**Keywords:** gastric cancer, ferroptosis, long non-coding RNA, prognostic model, tumor microenvironment, immunotherapy

## Abstract

**Background:** The construction of ferroptosis-related lncRNA prognostic models in malignancies has been an intense area of research recently. However, most of the studies focused on the exact expression of lncRNAs and had limited application values. Herein, we aim to establish a novel prognostic model for gastric cancer (GC) patients and discuss its correlation with immune landscapes and treatment responses.

**Methods:** The present study retrieved transcriptional data of GC patients from the Cancer Genome Atlas (TCGA) database. We identified differentially expressed ferroptosis-related lncRNAs between tumor and normal controls of GC samples. Based on a new method of cyclically single pairing, we constructed a 0 or 1 matrix of ferroptosis-related lncRNA pairs (FRLPs). A risk score signature consisting of 10 FRLPs was established using multi-step Cox regression analysis. Next, we performed a series of systematic analyses to investigate the association of the FRLP model and tumor microenvironment, biological function, and treatment responses. An alternative model to the FRLP risk score signature, the gene set score (GS) model was also constructed, which could represent the former when lncRNA expression was not available.

**Results:** We established a novel prognostic signature of 10 ferroptosis-related lncRNA pairs. High-risk patients in our risk score model were characterized by high infiltration of immune cells, upregulated carcinogenic and stromal activities, and heightened sensitivity to a wide range of anti-tumor drugs, whereas low-risk patients were associated with better responses to methotrexate treatment and elevated immunotherapeutic sensitivity. The practicability of the FRLP risk score model was also validated in two independent microarray datasets downloaded from Gene Expression Omnibus (GEO) using the GS model. Finally, two online dynamic nomograms were built to enhance the clinical utility of the study.

**Conclusion:** In this study, we developed a ferroptosis-related lncRNA pair-based risk score model that did not rely on the exact lncRNA expression level. This novel model might provide insights for the accurate prediction and comprehensive management for GC patients.

## Introduction

Gastric cancer (GC) is one of the most common malignancies worldwide. In consideration of its frequently advanced stage at diagnosis, GC is also the fourth leading cause of cancer-related deaths, with 768,793 deaths globally in 2020 (Sung et al., 2021). In China, GC remains the second most deadly cancer. According to statistics, there were 679,100 new GC cases and approximately 498,000 deaths occurred in 2015 (Chen et al., 2016). Currently, surgical resection is still the first-line treatment for GC patients, and the administration of adjuvant or neoadjuvant chemotherapy and immunotherapy could further improve patients’ prognosis. Unfortunately, resistance to chemotherapy occurs frequently, which remains a major cause of GC metastasis or relapse. Besides, the effect of immunotherapy is quite uncertain for GC patients due to the highly variant tumor microenvironment (TME) (Coutzac et al., 2019). Recent studies have linked ferroptosis with immune factors and chemotherapy resistance. Thus, it is necessary to further explore such an association in detail in GC.

Ferroptosis was firstly proposed by Dixon et al., (2012) and was described as an iron-dependent non-apoptotic cell death triggered by the small molecule erastin. The team summarized in another essay that ferroptosis was associated with reactive oxygen species production and lipid peroxidation (Dixon, 2017). In the past few years, ferroptosis has been implicated in multiple diseases and functions as a tumor suppression mechanism (Stockwell et al., 2020). Exploring the mechanisms underlying susceptibility and resistance to ferroptosis in carcinogenesis has been an intense area of research in the past few years (Friedmann Angeli et al., 2019). It was revealed by some studies that some tumor suppressor genes exert their anti-tumor function by upregulating tumor cells’ sensitivity to ferroptosis. For example, p53 (Wang et al., 2016) and BAP1 (Zhang et al., 2018) were found to downregulate the expression of SLC7A11, a negative modulator of ferroptosis. It was thus hypothesized that these two tumor-suppressive genes executed their anti-tumor function partly by enhancing cancer cells’ sensitivity to ferroptosis. Conversely, carcinoma’s resistance to ferroptosis was also reported to be connected with some of the malignant signatures in tumorigenesis such as hypoxia (Luis et al., 2021), EMT (Behan et al., 2019), and stemness (Wang et al., 2020). Studies have already highlighted the possibility for ferroptosis to be a novel target for cancer treatment (Liang et al., 2019; Koppula et al., 2021). When it comes to gastric cancer, the induction of ferroptosis has also been achieved in several studies (Hao et al., 2017; Chen et al., 2020; Cai et al., 2021; Zhao et al., 2021). These findings may provide new insights into ferroptosis-mediated cancer treatment.

Long non-coding RNAs (lncRNAs) are defined as non-protein coding transcripts of over 200 nucleotides. It was revealed that lncRNAs were aberrantly expressed in tumor tissues and were involved in the carcinogenesis of various cancer types (Bhan et al., 2017). Up to now, there have been some studies establishing lncRNA-based, ferroptosis-related risk signatures in relation to GC prognosis (Pan et al., 2021; Wei et al., 2021; Xiao et al., 2021; Zhang et al., 2022). Table 1 lists recent works that constructed ferroptosis-related lncRNA risk score models in GC. It is worth noting that some recent studies cyclically singly paired the ferroptosis-related lncRNAs and constructed prognostic risk score models of lncRNA pairs (Li et al., 2021; Tang et al., 2021). Compared with the aforementioned risk score model consisting of single lncRNA, these models did not rely on the specific expression of lncRNA and had broader clinical practicability, for it is unnecessary to transform data format when the expression profiles were obtained from different sequencing platforms.

**TABLE 1:** Recent Works Constructing Ferroptosis-related lncRNA Risk Score Models in GC patients.

Title	Risk score (RS) model			Hybrid model		Reference
	Number of lncRNAs	AUCs	Significance	Components	AUCs	
Construction of a ferroptosis-related lncRNA-based model to improve the prognostic evaluation of gastric cancer patients based on bioinformatics	17	0.750 for total OS	High-RS patients exhibited worse OS	Risk score	Not mentioned	Pan et al., (2021)
				Risk level		
				T stage		
				N stage		
				M stage		
				Age		
				Gender		
				Histological; Grade		
A novel ferroptosis-related lncRNA signature for prognosis prediction in gastric cancer	4	0.636 for total OS	High-RS patients exhibited worse OS; the four lncRNAs were validated to be aberrantly expressed in GC tumor tissues by RT-qPCR	Individual expression level of three lncRNAs; Risk score	Not mentioned	Wei et al, (2021)
A ferroptosis-related lncRNAs signature predicts prognosis and therapeutic response of gastric cancer	17	0.811 for total OS; 0.809 for 1-year OS; 0.805 for 3-year OS; 0.776 for 5-year OS	High-RS patients exhibited worse OS, higher tumorigenic events, lower gene mutation rates, and decreased sensitivity to anti-PD-L1 treatment; analysis in pan-cancer cell lines revealed that the RS was associated with IC50s of 41 anti-tumor drugs	Not constructed		Xiao et al, (2021)
Establishment and validation of a ferroptosis-related long non-coding RNA signature for predicting the prognosis of stomach Adenocarcinoma	3	0.660 for 3-year OS; 0.756 for 5-year OS	High-RS patients exhibited worse OS, advanced TNM stages, and increased tumorigenic events; the three lncRNAs were validated to be aberrantly expressed in GC cell lines by RT-qPCR	Risk score; TNM stage; age; gender	0.635 for 3-year OS; 0.661 for 5-year OS	Zhang et al., (2022)

In this study, we successfully established a novel ferroptosis-related lncRNA pair (FRLP) risk score model of clinical significance. An alternative gene set score (GS) model was also built to represent the FRLP model when the lncRNA expression profiles were not available. After confirming that the two models shared a high degree of compliance, we validated the FRLP risk score model in two external GEO cohorts using the GS model. Our present work could provide not only an accurate prediction for GC patients’ survival but also insightful strategies to optimize GC patient’ treatment.

## Materials and Methods

### Data Obtaining and Processing

The transcriptional data of 375 stomach adenocarcinoma (STAD) tissues and 32 normal tissues were downloaded from The Cancer Genome Atlas (TCGA; https://portal.gdc.cancer.gov) database. We also obtained the corresponding clinical data from the University of California Santa Cruz (UCSC; https://xena.ucsc.edu) database. Patients without clinical information were excluded. Overall, a total of 334 cases were included. The clinical information of patients, including age, gender, TNM status, stage, and tumor location, is listed in Table 2. Next, we transformed the Ensembl ID to gene symbols and picked out the lncRNAs using GTF annotation document downloaded from the ensemble human genome browser GRCh38. p13 (http://asia.ensembl.org/index.html). Ferroptosis-related genes were retrieved from the FerrDb (http://www.zhounan.org/) database.

**TABLE 2:** Characteristics of patients with STAD from TCGA database, GSE62254 and GSE84437.

Characteristics	TCGA (*n* = 334)	GSE62254 (*n* = 300)	GSE84437 (*n* = 433)
	No. of patients (%)	No. of patients (%)	No. of patients (%)
Age at diagnosis			
≤65	152 (45.5)	172 (0.6)	150 (0.3)
>65	182 (54.5)	128 (0.4)	283 (0.7)
Gender			
Male	217 (65)	199 (0.7)	296 (0.7)
Female	117 (35)	101 (0.3)	197 (0.3)
Tumor location			
Proximal	80 (24.0)	32 (0.1)	Not Available (NA)
Body/fundus	117 (35.0)	127 (0.4)	NA
Distal	123 (36.8)	155 (0.5)	NA
Others	14 (4.2)	6 (0.0)	NA
Tumor grade			
G1	198 (59.3)	NA	NA
G2	118 (35.3)	NA	NA
G3	9 (2.7)	NA	NA
unknown	9 (2.7)	NA	NA
Tumor stage			
Stage I	44 (13.2)	30 (0.1)	NA
Stage II	104 (31.1)	96 (0.3)	NA
Stage III	139 (41.6)	95 (0.3)	NA
Stage IV	34 (10.2)	77 (0.3)	NA
unknown	13 (3.9)	2 (0.0)	NA
T status			
T1	14 (4.2)	0 (0.0)	11 (0.0)
T2	72 (21.6)	186 (0.6)	38 (0.1)
T3	157 (47.0)	91 (0.3)	92 (0.2)
T4	87 (26.0)	21 (0.1)	292 (0.7)
unknown	4 (1.2)	2 (0.0)	0 (0.0)
N status			
N0	97 (29.0)	38 (0.1)	80 (0.2)
N1	91 (27.2)	131 (0.4)	188 (0.4)
N2	70 (21.0)	80 (0.3)	132 (0.3)
N3	66 (19.8)	51 (0.2)	33 (0.1)
unknown	10 (3.0)	0 (0.0)	0 (0.0)
M status			
M0	299 (89.5)	278 (0.9)	NA
M1	23 (6.9)	27 (0.1)	NA
unknown	12 (3.6)	0 (0.0)	NA

### Screening of Ferroptosis-Related lncRNAs

The frlncRNAs were identified by the Pearson correlation analysis (|R|>0.4 and *p* < 0.001). Next, we filtered out differentially expressed lncRNAs between tumor and normal controls using the “limma” package of R software under the criteria of |log fold change (FC)|> 2 and adjusted *p* value < 0.05. The volcano plot and heatmap of DEfrlncRNAs were depicted by the “ggplot2” and “pheatmap” packages of R software.

### LncRNA Pairing

All the DEfrlncRNAs were cyclically single-paired and a 0 or 1 matrix was established according to the following procedure (Luis et al., 2021): if the expression of lncRNA a was higher than its pairing lncRNA, lncRNA b, the value of this lncRNA pair was defined as 1; conversely, we regarded the pair’s value as 0 if lncRNA A’s expression was lower than lncRNA B. FrlncRNA pairs (FRLPs) were excluded if there were too many (more than 80%) 0 or 1 values. In other words, only the lncRNA pairs with an optimal 0 or 1 range (20%–80%) were sorted out for further analysis. Univariate Cox regression analyses were performed to evaluate the prognostic value of FRLPs (*p* < 0.01).

### Risk Score Model Construction

Using R package “glmnet”, the least absolute shrinkage and selection operator (LASSO) regression analysis was conducted in TCGA cohort, and the lncRNA pairs filtered out were further subjected to multivariate Cox regression analysis. The multicollinearity of lncRNA pairs was estimated through the variance inflation factor (VIFs), and we defined that VIF ≥2 was considered to indicate multicollinearity in the study (Yan et al., 2021). LncRNA pairs that did not violate the multicollinearity assumption were filtered out for model construction. Risk scores were calculated based on the formula below:

$$Risk\;score=\sum_{i=1}^{n}Coef\left(\ln cRNA\;pair\;i\right)*Val\left(\ln cRNA\;pair\;i\right)$$

Coef (lncRNA pair i) and Exp (lncRNA pair i) indicated the coefficient and value (0 or 1) of an individual lncRNA pair I, respectively. Time-dependent receiver operating characteristic (ROC) curve (Kamarudin et al., 2017) and decision curve analysis (DCA) (Vickers and Elkin, 2006) were employed to access the predictive efficacy of the FRLP risk score model. Then, patients were divided into high- and low-risk groups based on the maximum reflection point of the most suitable ROC curve. Comparisons of differences in overall survival (OS) were conducted using the Kaplan–Meier method.

### Development and Evaluation of Nomogram Based on the Risk Score

To quantitatively estimate our FRLP risk score model’s prognostic potential in clinical practice, we created a nomogram using “Survival” and “RMS” packages in R to predict 1, 3-, or 5-year OS. The nomogram integrated risk score and other clinicopathological variables, which were also identified as independent risk factors by the Cox regression analysis. In addition, using the “coxph” function of R package “survival,” we performed Schoenfield’s residual test to decide whether these risk factors met the equally proportional risk hypothesis. Finally, the robustness of the nomogram was also evaluated by the calibration curves, time-dependent ROC curves, and DCA analysis (Vickers and Elkin, 2006).

### Clinical Correlation Analysis

To explore the clinical significance of our risk score model, we analyzed the difference in the clinicopathological characteristics between the two risk groups by the chi-square test. The result was depicted in a heatmap form using R packages “pheatmap” and “ggpubr.” The clinicopathological characteristics include age, gender, tumor grade, tumor location, and TNM status. Moreover, we conducted a stratification analysis comparing the risk score difference in different clinical groups: age (>65 and ≤65), gender (male and female), tumor stage (I–II and III–IV), grade (G1–G2 and G3), tumor location (proximal, body/fundus and distal), T status (T1–T2 and T3–T4), N status (N0 and N1–N3), and M status (M0 and M1).

### Somatic Variation Analysis

Using VarScan2 annotation files downloaded from TCGA database, the tumor mutation burden (TMB) of each sample was calculated through the VarScan2 pipeline somatic mutation calling workflow (Binatti et al., 2021). A comparison of TMB between the two groups was carried out. Survival analysis combining the risk score and TMB was performed. Spearman correlation analysis was performed to test the relation between TMB and risk scores. The top 20 genes with the highest mutation frequencies were visualized using the “maftools” package in R.

### Tumor Microenvironment Analysis

The stromal and immune cell content in the microenvironment was quantified for every STAD patient using the “ESTIMATE” algorithm, and a comparison was performed between the two risk groups. Next, several currently acknowledged algorithms include TIMER, xCell, quanTIseq, MCP-counter, EPIC, CIBERSORT-ABS, and CIBERSORT. to calculate the contents of tumor infiltrating immune cells (TIICs). The whole analysis process was performed *via* the online platform Tumor Immune Estimation Resource 2.0 (TIMER2.0, http://timer.cistrome.org/). Spearman correlation analysis was further performed to analyze the correlations between the risk score and immune cells. We set “R > 0.1, *p* < 0.05” as the criterion and visualized our results in the form of a lollipop diagram using the R package “ggplot2.”

### Immunotherapeutic Sensitivity Analysis

To explore the correlation between risk score and response toward the immune checkpoint blockage (ICB) treatment, we obtained 24 immune checkpoint genes (ICGs) from previous literatures (Garris et al., 2018; Han et al., 2019; Nishino et al., 2017; Patel et al., 2017; Yang et al., 2017). The tumor immune dysfunction and exclusion (TIDE) score was defined by Jiang and his colleagues (Jiang et al., 2018) and has been proved to be a reliable indicator for predicting the ICB treatment response. We obtained the TIDE score, dysfunction score, and exclusion score of each patient from the TIDE website (http://tide.dfci.harvard.edu/). In addition, the immunophenoscore (IPS), calculated by unbiased machine learning methods, is another parameter reflecting immunogenicity. Higher IPS represents better accuracy for the more corresponding result (Jiang et al., 2021). The IPS results for anti-CTLA4 and anti-PD1 treatments of TCGA STAD patients were downloaded from The Cancer Immunome Atlas (TCIA, https://tcia.at/home). Two groups’ differences in IPS and TIDE scores were compared.

### Biological Function Analysis

To shed light on the difference in biological functions between the two risk groups, “KEGG” gene sets “c2. cp.kegg.v7.0. symbol.gmt” and “GO” gene sets “c5. go.v7.4. symbols.gmt” were downloaded from the MsigDB (http://www.gsea-msigdb.org) database for gene set enrichment analysis (GSEA) (Subramanian et al., 2005). Besides, “Hallmark” gene set “h.all.v7.4. symbols.gmt,” which contains 50 well-defined biological signatures, were also available from MsigDB (Subramanian et al., 2005). We estimated the enrichment score (ES) of each signature using single sample gene set enrichment analysis (ssGSEA).

### Chemotherapy Response Analysis

Half-inhibitory concentration (IC50) of different types of chemotherapeutic drugs was estimated using R package “pRRophetic.” The “pRRophetic” package selected 138 kinds of drugs from more than 700 cell lines in the Genomics of Drug Sensitivity in Cancer database (GDSC, https://www.cancerrxgene.org/) and developed a ridge regression algorithm to predict treatment responses. Here, we only chose gastrointestinal cell lines to ensure a more accurate prediction by setting the parameter “tissueType” of the “pRRopheticPredict” function as “digestive_system.” We compared the difference of IC50, and the results were displayed in the form of boxplots using R package “ggpubr.”

### External Validation of the FRLP Risk Score Model

Two microarray datasets for GC patients, GSE84437 and GSE62254, were downloaded from the Gene Expression Omnibus (GEO, https://www.ncbi.nlm.nih.gov/geo) database and used for external validation of the FRLP risk score model. The clinical information of patients from these two cohorts is also listed in Table 2. An alternative model to the FRLP risk score, the GS model was established and used to conduct the external validation (Zhang et al., 2021). Differentially expressed genes (DEGs) between high- and low-risk score groups were screened out based on |logFC|>0.585 (|FC|>1.5), *p* < 0.05 criteria. Gene set A comprised DEGs upregulated in the high-risk group, while low-risk group’s highly expressed DEGs were classified into gene set B. We performed ssGSEA to calculate the enrichment score (ES) of gene sets A and B in each sample. Consequently, the gene set score (GS), namely the subtraction of gene set B ES from gene set A ES, was estimated and used to represent the risk score when lncRNA expression profiles were not available. We performed the Spearman correlation analysis to show the relevance of the two scores (Zhang et al., 2021). Using the cut-off of the ROC curves for GS model, we classified patients into high- and low-risk groups. Then, we compared the patients’ stratification between the FRLP risk score model and the alternative GS model. A Sankey diagram was drawn to demonstrate the intersection of the two models using R package “Director” (Icay et al., 2018; Xiang et al., 2021; Tang et al., 2021). Finally, we calculated the GS of each sample in two GEO cohorts and stratified the patients using the same cut-off value mentioned above. We analyzed the differences in survival, TIDE score, and drug sensitivity between high- and low-risk groups. Additionally, a nomogram based on the GS model was constructed to enhance the clinical utility.

### Development of Dynamic Nomograms Based on FRLP Risk Score Model and GS Model

To further enhance the clinical utility of the two aforementioned nomograms, we sought to visualize them in an interactive form. Using R package “DynNom” (Jalali et al., 2019; Yin et al., 2022), we generated two dynamic nomograms with interactive interfaces for clinical application based on the FRLP risk score model and GS model, respectively.

### Statistical Analysis

All statistical analyses were performed in R version 4.1.2. Wilcoxon test was performed to conduct numerical difference comparisons of two groups. Log-rank test was performed to evaluate the differences in survival, and Kaplan–Meier plots were drawn to visualize the comparison. Univariate and multivariate Cox proportional hazard regression analyses were used to assess the predictive efficacy of the risk score. Statistical significance was set at *p* < 0.05.

## Results

### Identification of FRLPs in TCGA Cohort

**FIGURE 1:**
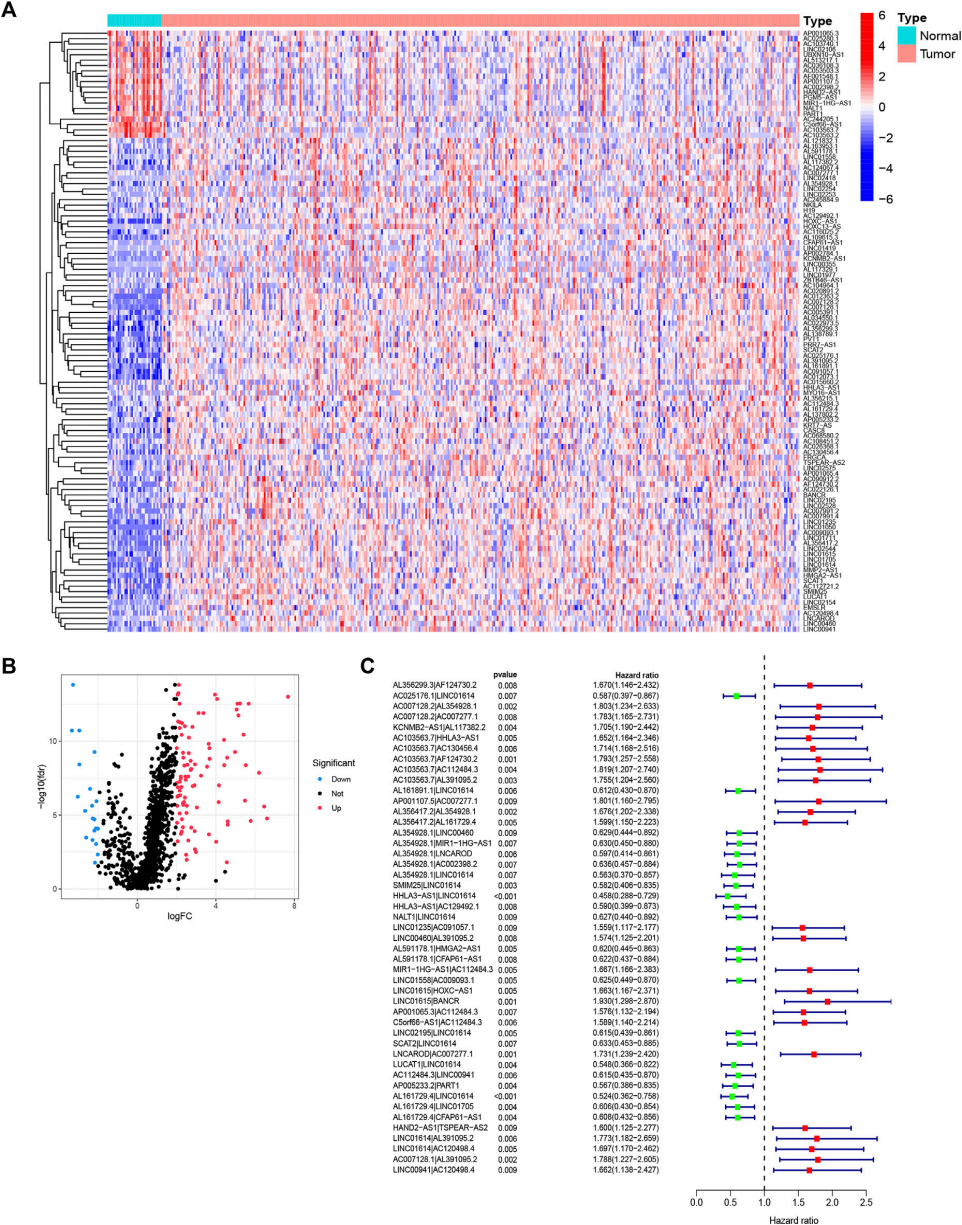
Identification of differentially expressed ferroptosis-related lncRNAs (DElncRNAs) and differentially expressed ferroptosis-lncRNA pairs (FRLPs). **(A)** Heat map of 112 DElncRNAs. Red dots and blue dots represent upregulated and downregulated lncRNAs in tumor samples, respectively. **(B)** Volcano map of 112 DElncRNAs. **(C)** Forest plot showing 47 prognostic FRLPs identified by univariate Cox analysis.

The entire analytical process of the study is presented in Supplementary Figure S1. Our study included the transcriptome data of 375 STAD samples and 32 normal samples from the TCGA database and identified 14,086 lncRNAs. A total of 382 ferroptosis-related genes were downloaded from the FerrDb, and their expression was analyzed in the TCGA STAD expression matrix. We conducted the Pearson correlation analysis and screened out 1,343 ferroptosis-related lncRNAs. Then, 112 differentially expressed lncRNAs (DELs) were filtered out according to the |logFC|>2 criteria, of which 92 were upregulated and 20 were downregulated in tumor samples, respectively (Figures 1A,B). After pairing analysis of the 112 DELs, a 0 or 1 matrix of 4,722 FRLPs was established. Finally, the univariate Cox analysis identified 47 prognostic FRLPs (Figure 1C).

### Construction of FRLP Risk Score Model

**FIGURE 2:**
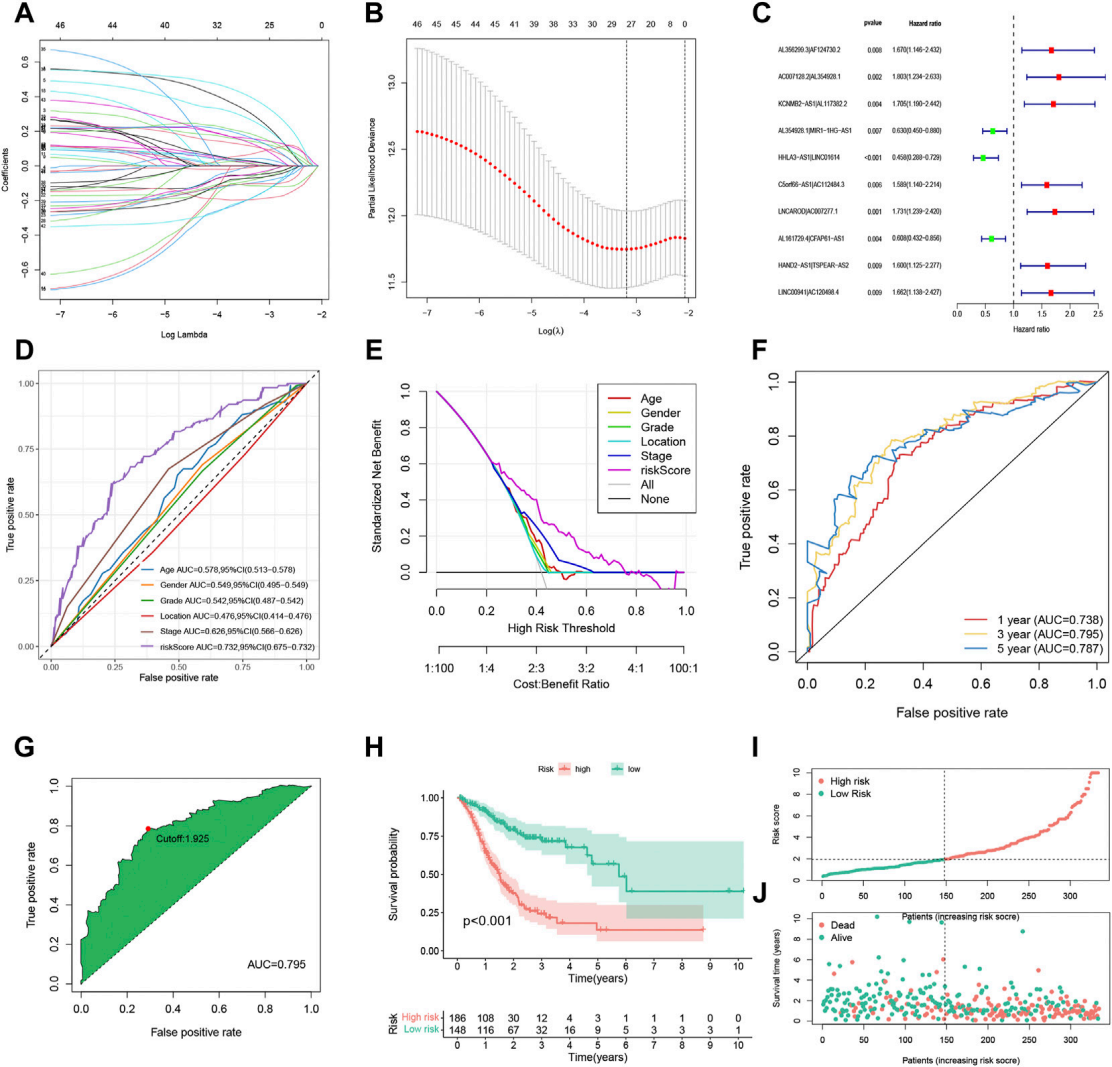
Construction and evaluation of the FRLP risk score model in TCGA cohort. **(A)** Least absolute shrinkage and selection operator (LASSO) coefficients of 27 prognosis-related FRLPs. **(B)** Tenfold cross-validation for tuning parameter selection in the LASSO model. **(C)** Forest plot showing 10 FRLPs identified by multivariate Cox regression analysis. **(D)** Receiver operating characteristic (ROC) comparing the risk score and other clinical factors in predicting total OS. **(E)** Decision curve analysis (DCA) curves estimating the predictive efficacy of the risk score from the perspective of clinical benefit. The *y*-axis refers to the net benefit. The x-axis refers to the predicted OS. The black line represents the hypothesis that all patients survive in 5 years. The gray line represents the hypothesis that no patients stay alive for more than 1 year. **(F)** ROC curve for predicting 1-, 3-, and 5-year overall survival (OS) of the FRLP risk score model. **(G)** Cut-off point of the risk score model. **(H)** Kaplan–Meier plot of high- and low-risk patients. **(I)** The risk score distribution. Green dots represent risk scores for low-risk patients; red dots represent risk scores for high-risk patients. **(J)** The relationship between survival status and risk score. The horizontal ordinate represents the number of patients; the vertical ordinate represents risk score (AUC: area under curve; CI: confidence interval).

To further explore the prognostic value of FRLPs in GC, a stepwise Cox regression procedure was performed. Firstly, to reduce the overfitting among 46 prognostic FRLPs, we conducted a LASSO Cox regression analysis and 27 FRLPs were filtered out according to the minimum partial likelihood deviance (Figures 2A,B). Then, we performed multivariate Cox analysis on these 27 FRLPs and finally obtained 10 prognostic FRLPs (Figure 2C). The list of 10 FRLPs and their corresponding calculation coefficients are shown in Table 3. As VIFs were all <2, there wasn’t any multicollinearity relationship between these FRLPs (Table 4). Compared with other clinical factors, our risk score model was more accurate to predict the overall survival according to multi-factor ROC analysis (Figure 2D). Then, we conducted DCA to explore the clinical significance of the risk score. As shown in Figure 2E, the risk score displayed better net benefit than other factors, which indicated that the risk score was competent to help clinicians make more accurate assessment of patient prognosis compared with the age-, gender-, grade-, location-, or stage-only model. Moreover, the time-dependent ROC analysis revealed that the area under curves (AUCs) for 1-, 3-, and 5-year overall survival (OS) were 0.738, 0.795, and 0.787, respectively (Figure 2F).

**TABLE 3:** lncRNA pairs used for the construction of the FRLP risk score model.

lncRNA pairs	Coefficient
AL356299.3|AF124730.2	0.545
AC007128.2|AL354928.1	0.505
KCNMB2-AS1|AL117382.2	0.448
AL354928.1|MIR1-1HG-AS1	−0.447
HHLA3-AS1|LINC01614	−0.580
C5orf66-AS1|AC112484.3	0.301
LNCAROD|AC007277.1	0.616
AL161729.4|CFAP61-AS1	−0.401
HAND2-AS1|TSPEAR-AS2	0.380
LINC00941|AC120498.4	0.410

**TABLE 4:** The variance inflation factors (VIFs) of 10 lncRNA pairs.

lncRNA pairs	VIFs
AL356299.3|AF124730.2	1.048
AC007128.2|AL354928.1	1.063
KCNMB2-AS1|AL117382.2	1.049
AL354928.1|MIR1-1HG-AS1	1.424
HHLA3-AS1|LINC01614	1.047
C5orf66-AS1|AC112484.3	1.079
LNCAROD|AC007277.1	1.058
AL161729.4|CFAP61-AS1	1.064
HAND2-AS1|TSPEAR-AS2	1.465
LINC00941|AC120498.4	1.06

### Predictive Assessment of the FRLP Risk Score Model

**FIGURE 3:**
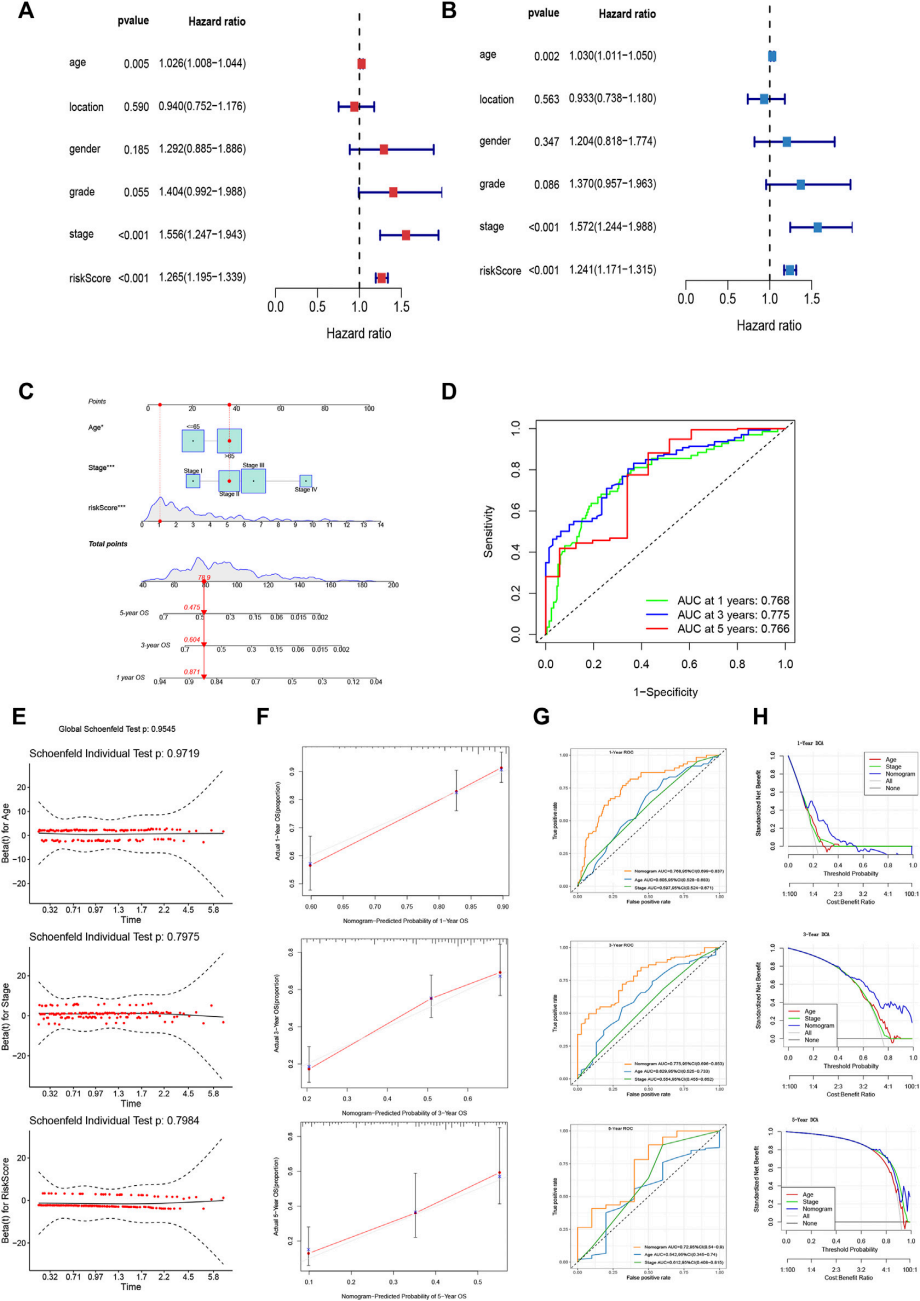
Evaluation of FRLP risk score model’s predictive efficacy. **(A)** Univariate Cox analysis of risk score and other clinicopathological factors. **(B)** Multivariate Cox analysis of risk score and other clinicopathological factors. **(C)** Nomogram integrating risk score, age, and tumor stage for predicting 1-, 3-, and 5-year OS. **(D)** Time-dependent ROC curve of the nomogram for predicting 1-, 3-, and 5-year OS. **(E)** Schoenfield’s residual test of age, stage, and risk score. **(F)** Calibration curves of the nomogram for predicting 1-, 3-, and 5-year OS. The gray lines represent the ideal predictive model, and the red lines represent the observed model. **(G)** Time-dependent ROC curves evaluating the efficacy of the nomogram to predict 1-, 3-, and 5-year OS. **(H)** DCA curves estimating the predictive efficacy of the nomogram from the perspective of clinical benefit.

We identified the maximum inflection point of 1.925 as the optimal cut-off point on the 3-year ROC curve (Figure 2G). Subsequently, 148 patients were classified into the low-risk group and 186 into high-risk group. The OS of the low-risk group was significantly longer than that of the high-risk group (Figure 2H). The distribution of patients’ risk score is depicted in Figure 2I. Based on the scatter plot, the number of deaths increased as the risk score increased (Figure 2J). To figure out whether the risk score model is independent of other clinicopathological parameters, univariate and multivariate Cox analyses incorporating age, gender, tumor location, and tumor stage were adopted. It was revealed that the FRLP model is an independent prognostic factor for STAD patients (univariate Cox *p* < 0.001, HR = 1.265, 95% CI 1.195–1.339; multivariate Cox *p* < 0.001, HR = 1.241, 95% CI: 1.171–1.315; Figures 3A,B). Subsequently, we built a nomogram that integrated tumor stage and risk score to predict patients’ 1-, 3-, and 5-year OS (Figure 3C). The AUCs of the nomogram for predicting 1-, 3-, and 5-year OS rates were 0.768, 0.775, and 0.766, respectively (Figure 3D). According to Schoenfield’s residual test, the individual *p*-value for age, stage, and risk score was 0.97, 0.80, and 0.80, respectively, while the global *p*-value was 0.95 (Figure 3E). These results indicated that the nomogram model met the equally proportional risk hypothesis. The total point of the nomogram (nomoscore) had higher efficacy in predicting GC patients’ 1, 3, and 5-year OS than age or stage (Figure 3F). Moreover, DCA analysis also revealed that the nomoscore displayed better net benefit than the age- or stage-alone model (Figure 3G). The calibration curve also confirmed the predictive value of the nomogram in predicting 1-, 3-, and 5-year OS (Figure 3H).

### The Clinical Significance of the FRLP Risk Score Model

**FIGURE 4:**
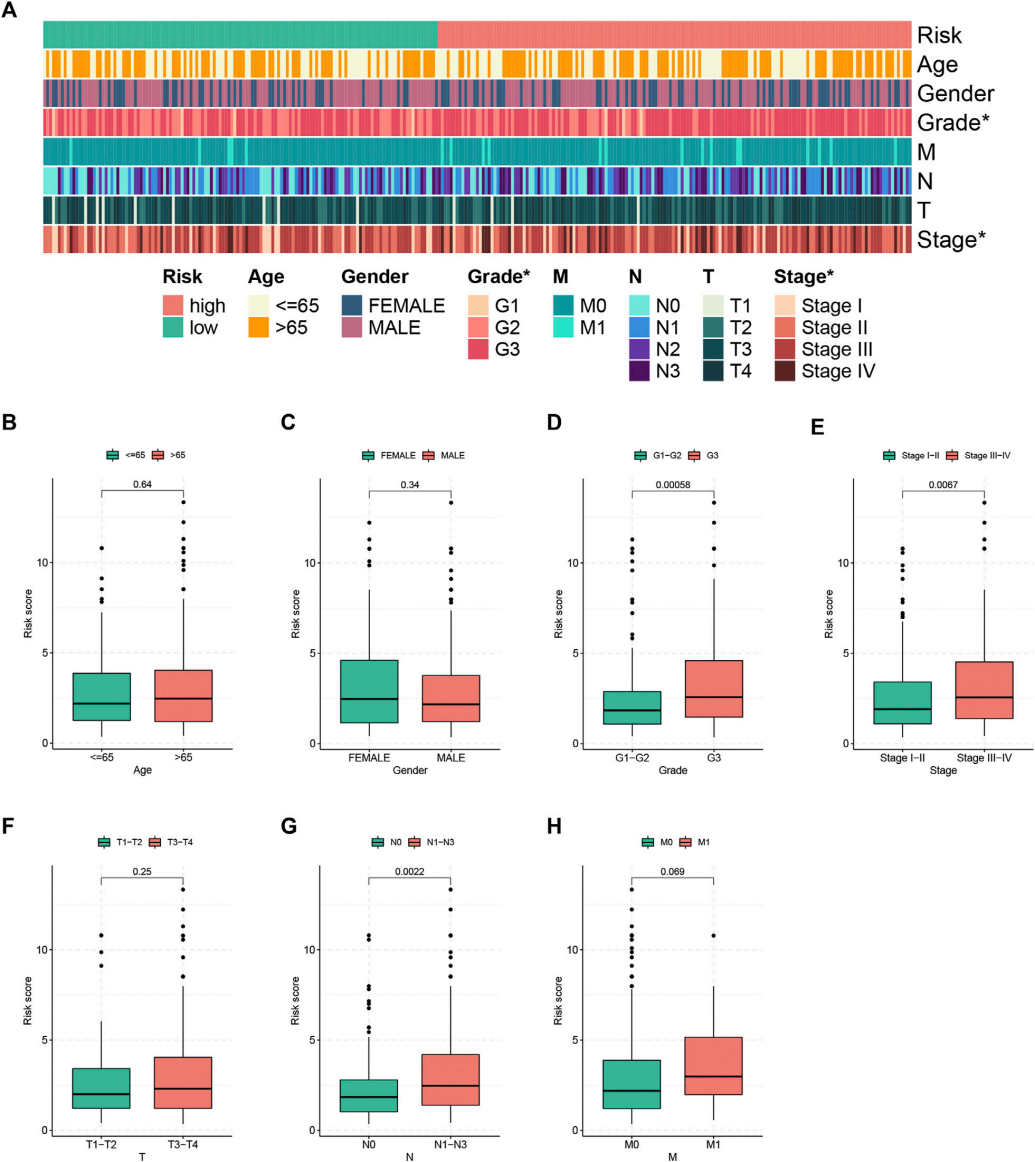
Clinical correlation of the FRLP risk score model. **(A)** Heatmap showing the clinical relevance of the risk score model (*p < 0.05). **(B–H)** Boxplots showing risk score differences in different age, gender, tumor grade, tumor stage, T status, N status, and M status groups.

To evaluate the relationship risk score and clinicopathological characteristics, chi-square test was performed and the results are demonstrated in Figure 4A, which indicated that tumor grade and tumor stage were closely linked to the FRLP risk level. In addition, we analyzed the risk score differences between groups stratified by different clinicopathological factors. As shown in Figures 4B–H, a high-risk score with statistical significance was more common to see in patients with higher tumor grades as well as more advanced N stages and TNM stages. Nevertheless, patients in different gender, age, M status, and T status groups exhibited no differences in risk scores.

### The Correlation of the FRLP Risk Score Model and Somatic Variance

**FIGURE 5:**
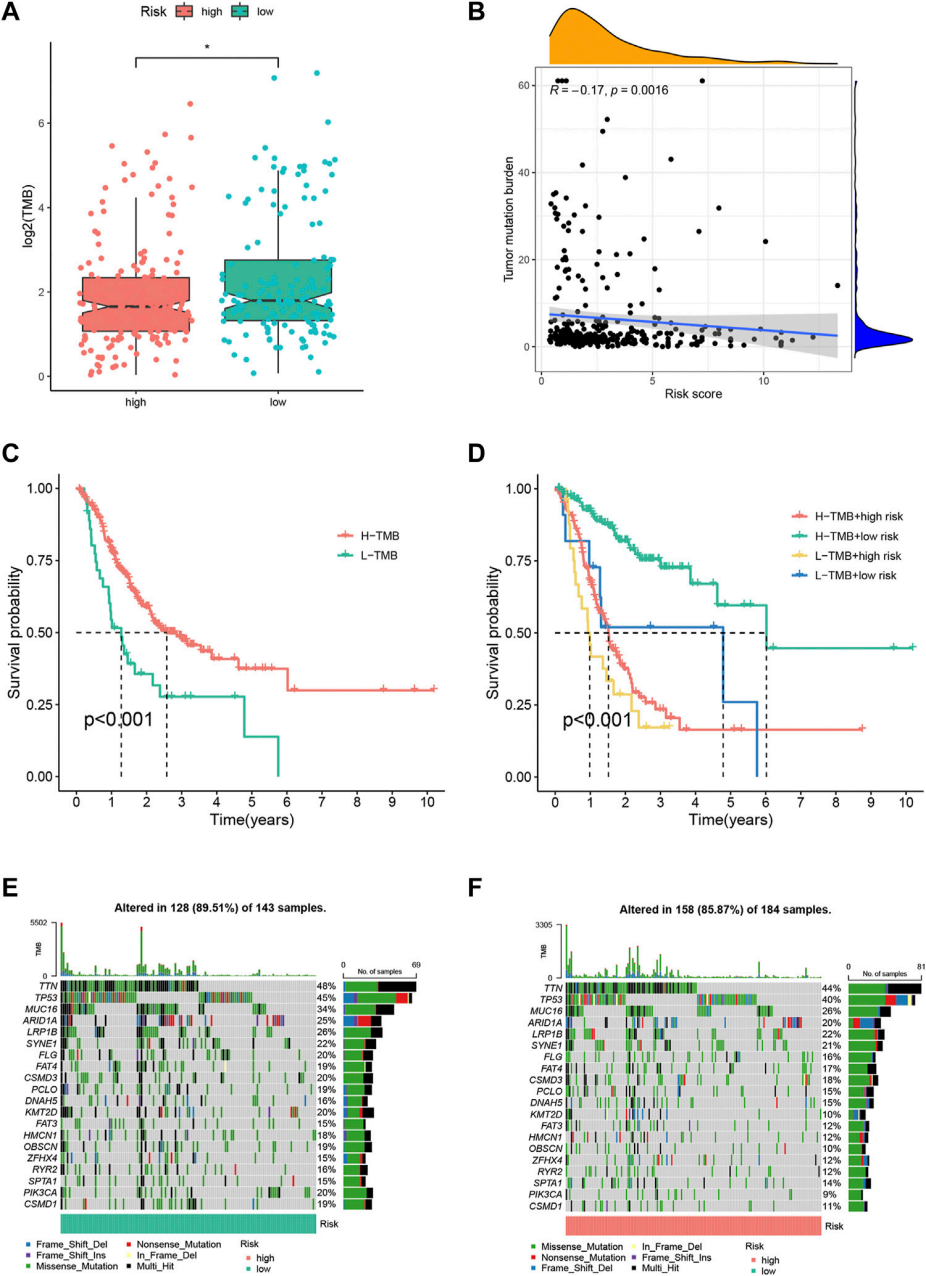
Tumor mutation burden (TMB) analysis of the FRLP risk score model. **(A)** TMB difference between high- and low-risk groups. **(B)** Correlation between the risk score and TMB. **(C)** Kaplan–Meier plots of patients with high and low TMB. **(D)** Kaplan–Meier curves of patients stratified by both TMB and the risk score. **(E,F)** Gene mutation analysis of patients in low- and high-risk groups.

To investigate the gene mutation landscape of the FRLP risk score model, we performed TMB and gene mutation frequency analysis. As shown in Figure 5A, low-risk STAD patients had higher TMB levels than high-risk patients. And a negative correlation between TMB levels and risk score was found according to the Spearman correlation analysis (R = −0.17, *p* = 0.0016) (Figure 5B). The Kaplan–Meier analysis suggested that STAD patients with high TMB had longer OS than low TMB patients (Figure 5C). Stratified survival analysis further confirmed that the survival benefits for high TMB patients still exist in both high- and low-risk groups (Figure 5D). In general, gene mutation frequency was higher in the low-risk group than that in the high-risk group (Figures 5E,F).

### The Correlation of the FRLP Risk Score Model and Tumor Microenvironment

**FIGURE 6:**
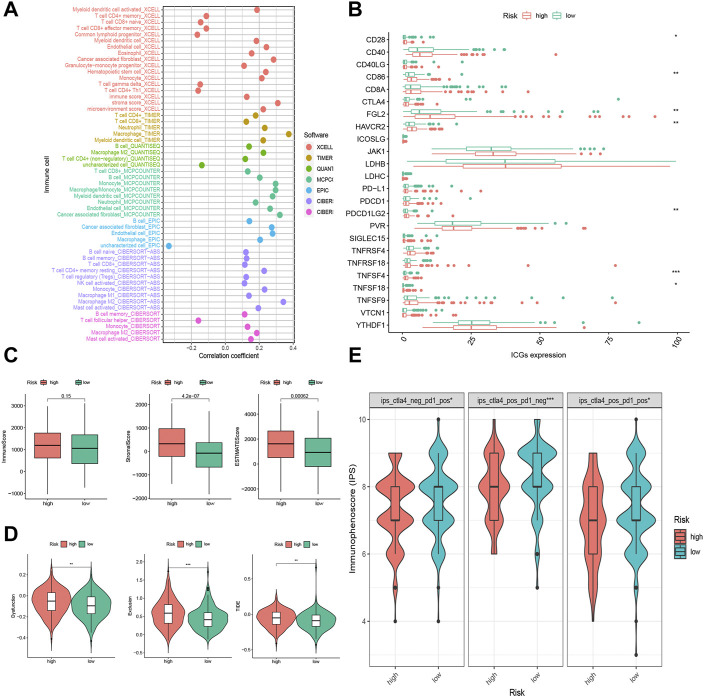
Tumor infiltrating immune cells (TIICs) and immunotherapeutic sensitivity analysis of the FRLP risk score model. **(A)** The correlation between risk score and TIICs analyzed by seven different quantification methods of immune infiltration estimations including TIMER, xCell, quanTIseq, MCP-counter, EPIC, CIBERSORT-ABS, and CIBERSORT. **(B)** Expression of 24 immune checkpoint genes in high- and low-risk groups. **(C)** Boxplots showing immune score, stromal score, and ESTIMATE score in high- and low-risk groups. **(D)** Boxplots showing dysfunction score, exclusion score, and tumor immune dysfunction and exclusion (TIDE) score differences between high- and low-risk score groups. **(E)** Immunophenoscore (IPS) differences for ICB treatment between high- and low-risk groups; ips_ctla4_neg_pd1_pos refers to CTLA4-negative response and PD1-positive response; ips_ctla4_pos_pd1_neg refers to CTLA4-positive response and PD1-negative response; ips_ctla4_pos_pd1_pos refers to CTLA4-positive response and PD1-positive response (∗*p* < 0.05, ∗∗*p* < 0.01, ∗∗∗*p* < 0.001).

To shed light on the model’s association with tumor microenvironment, we estimated the stromal scores, immune scores, and ESTIMATE scores of two risk groups by the R package “ESTIMATE”. These three scores were reflections of stromal contents, immune cell contents, and the aggregation of the two contents. Then, we calculated the content of tumor infiltrating immune cells (TIICs) using diversified algorithms online and discussed the correlation between the risk score and tumor-infiltrating immune cells *via* Spearman correlation analysis. The results demonstrated that the stromal score and ESTIMATE score were higher in the high-risk group, while there was no significant difference in the immune score between the two groups (Figure 6C). High-risk scores were more closely linked with high TIICs (Figure 6A). We specified the MCPcounter algorithm result, and it was indicated that immunosuppressive cells (e.g., endothelial cells, fibroblasts, monocytes, and neutrophils) were more densely infiltrated in the high-risk group, whereas there was no statistical significance of anti-tumor immune cells (e.g., B lineage, CD8 T cells, cytotoxic lymphocytes, NK cells, and T cells) infiltration between the two groups (Supplementary Figure S1).

### The Correlation of the FRLP Risk Score Model and Immunotherapeutic Sensitivity

Of all the 24 selected ICGs, CD28, CD86, FGL2, HAVCR2, PDCD1LG2, TNFSF4, and TNFSF18 were highly expressed in the high-risk group (Figure 6B). These results further confirmed the immunosuppressive phenotype of the high-risk patients. Intriguingly, according to the TIDE analysis, which suggested that high-risk patients had a heightened level of dysfunction score, exclusion score, and TIDE score (Figure 6D), high-risk patients might not actually benefit from ICB treatment though highly expressed in ICGs. On the contrary, the IPS scores of anti-CTLA4+ anti-PD1+, anti-CTLA4- anti-PD1+, and anti-CTLA4+ anti-PD1- were all higher in low-risk subgroups (Figure 6E), implying low-risk patients’ better responses toward anti-CTLA4 and/or anti-PD1 immunotherapy.

### The Correlation of the FRLP Risk Score Model and Biological Function

**FIGURE 7:**
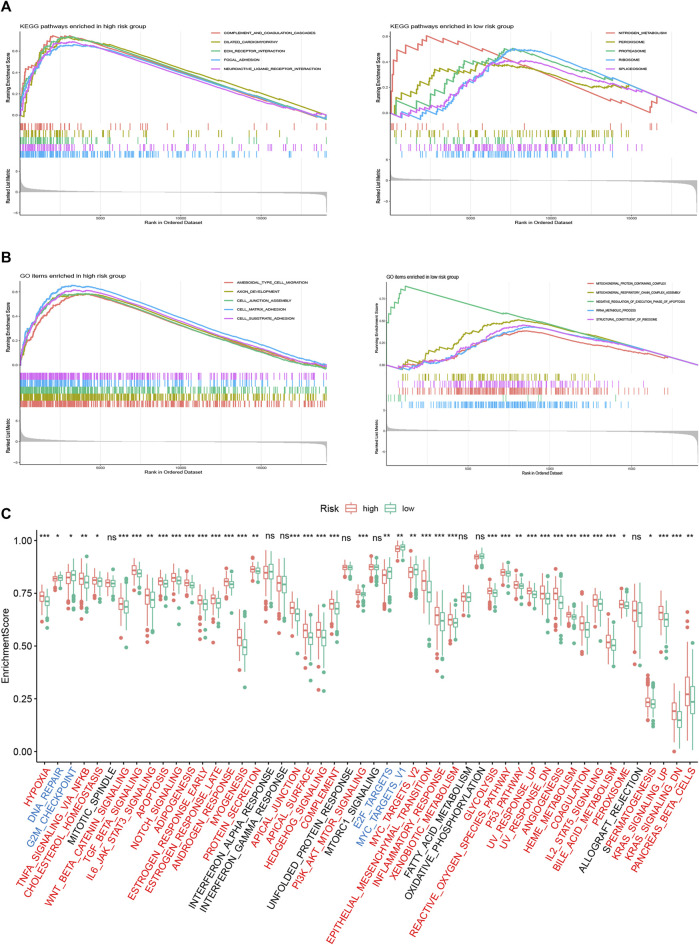
Biological function analysis of the FRLP risk score model. **(A)** Gene set enrichment analysis (GSEA) analysis using KEGG gene sets for high- and low-risk groups. **(B)** GSEA analysis using GO gene sets for high- and low-risk groups. **(C)** ssGSEA analysis using Hallmark gene sets for high- and low-risk groups. Gene sets markedly enriched in high- or low-risk groups were marked red and blue, respectively (∗*p* < 0.05, ∗∗*p* < 0.01, ∗∗∗*p* < 0.001, ns: not significantly).

To investigate different risk groups’ enriched biological function, GSEA using “KEGG” and “GO” gene sets was conducted to compare the enrichment differences between two risk groups. As shown in Figures 7A,B, KEGG pathways in relation to stromal activity and diseases including “ECM–receptor interaction,” “focal adhesion,” and “dilated cardiomyopathy” were enriched in the high-risk group. Meanwhile, GO items in relation to cell migration such as “ameboidal-type cell migration,” “cell junction assembly,” and “cell matrix adhesion” were enriched in the high-risk group. For the low risk group, we found an enrichment tendency toward KEGG metabolism-related pathways (e.g., “nitrogen metabolism,” “ribosome,” and “peroxisome”) and GO metabolism-related items (e.g., “rRNA metabolic process,” “mitochondrial-protein containing complex,” and “structural constituent of ribosome”). In addition, we performed ssGSEA using the “Hallmark” gene sets. As revealed by Figure 7C, the high-risk group was markedly correlated with carcinogenic activities (e.g., “Wnt beta catenin,” “myc targets,” “kras signaling,” etc.) and stromal pathways (e.g., “hypoxia,” “angiogenesis,” and “epithelial–mesenchymal transition”), whereas the low-risk group was characterized by its enrichment in cell cycle-related events (e.g., “DNA repair,” “G2M checkpoint,” and “E2F targets”).

### External Validation of the FRLP Risk Score Model

The lncRNAs in our FRLP risk score model could not be detected in two GEO microarray datasets due to limited sequencing depth. Therefore, we constructed gene set A and gene set B, which were composed of genes significantly upregulated in high- and low-risk groups, respectively (Supplementary Figure S3A, Supplementary Table S1). KEGG analysis suggested that the two gene sets targeted at different pathways, with gene set A associated with stromal and carcinogenic pathways and gene set B targeted at immune-related pathways. (Supplementary Figures S3B,C). Then, we developed a novel gene set score (GS) model that was defined as the subtraction of gene set B enrichment score (ES) from gene set A ES. The distribution of GS and the ES of two gene sets was significantly between high- and low-FRLP risk score groups, and the high-FRLP risk score group also exhibited a higher GS score (Supplementary Figure S3D). Moreover, there was a positive correlation between risk score and GS score (Spearman correlation R = 0.57; Supplementary Figure S3E). Subsequently, patients were divided into high- and low-GS groups according to the cut-off value of 3-year ROC curves, which was identical to the method we used to construct the FRLP risk score model (Supplementary Figure S3F). There was a significant overlap between the established FRLP risk score model and the GS model: over 70% of the high-FRLP risk score patients could be classified into a high-GS group, and the proportion of low-FRLP risk score patients falling to the category of low-GS group is over 75% (Supplementary Figures S3G,H). Since the GS model had a high degree of compliance with the FRLP risk score model, it is reasonable to regard the GS score as an alternative to FRLP risk score in distinguishing GC patients’ survival, biological function, tumor microenvironment, etc.

**FIGURE 8:**
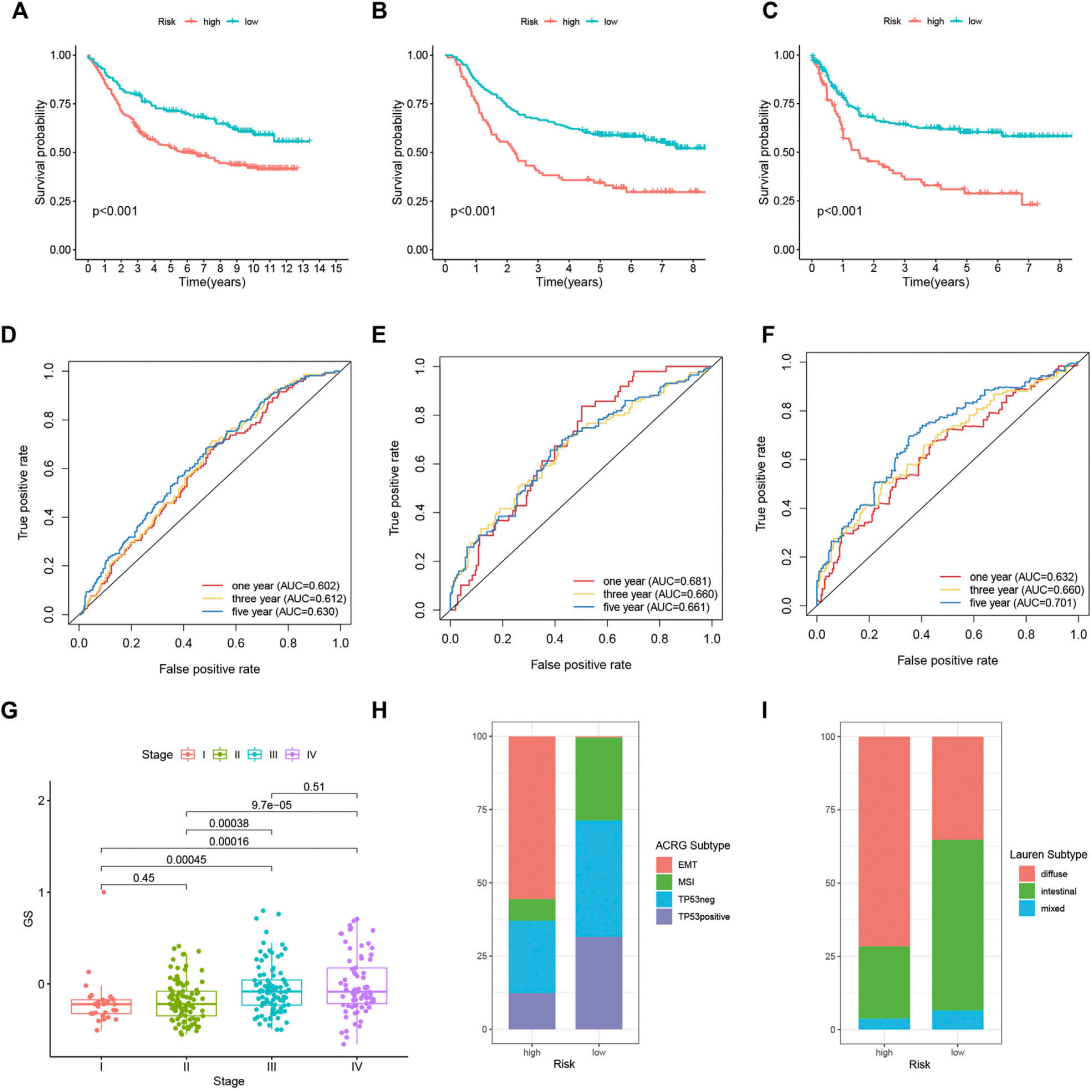
External validation of the FRLP risk score model in two GEO cohorts using the alternative GS model. Kaplan–Meier plots comparing OS of high- and low-risk patients in GSE84437 **(A)** and GSE62254 **(B)**. **(C)** Kaplan–Meier plots comparing disease-free survival (DFS) of high- and low-risk patients in GSE62254. ROC analysis of the GS model in predicting 1, 3, and 5 year OS in **(D)** GSE84437 and **(E)** GSE62254. **(F)** ROC analysis of the GS model in predicting 1, 3, and 5 year DFS in GSE62254. **(G)** Boxplots comparing GS score differences among different stages of patients in GSE62254. **(H,I)** Barplots showing the relative proportion of four Asian Cancer Research Group (ACRG) subtypes and three Lauren subtypes in high- and low-risk patients in GSE62254.

We calculated the GS of each sample in two GEO cohorts, and the classification of distinct risk groups was also conducted using the same cut-off value in the TCGA cohort (Supplementary Figure S3D). High-risk patients were validated to have worse OS in GSE84437 (*n* = 433, log rank *p* < 0.001), GSE62254 (*n* = 300, log rank *p* < 0.001), and shortened disease-free survival (DFS) in GSE62254 (log rank *p* < 0.001) (Figures 8A–C). Time-dependent ROC analysis also identified the efficacy of the alternative GS model in predicting patients’ survival. In GSE62254, the AUCs for 1-, 3-, and 5-year’s OS were 0.681, 0.660, and 0.661, respectively; the AUCs for 1-, 3-, and 5-year’s DFS were 0.632, 0.660, and 0.701, respectively. In GSE84437, the AUCs for 1-, 3-, and 5-year’s OS were 0.578, 0.612, and 0.630, respectively (Figures 8D–F). Specifically, in GSE62254, stage III/IV patients tended to exhibit higher GS (Figure 8G). Moreover, GSE62254 contains information of two classification systems for GC patients: the Asian Cancer Research Group (ACRG) subtype (Cristescu et al., 2015) and Lauren subtype. By inspecting the intersection of our GS model and these two subtypes, we found that there were a larger proportion of high-risk patients classified into “EMT” ACRG subtype and “diffuse” Lauren subtype (Figures 8H,I), which were both subtypes indicating worse clinical outcomes. In addition, TIDE analysis also showed elevated dysfunction, exclusion, and TIDE scores in high-risk groups in both of the two cohorts (Supplementary Figure S4).

### The Correlation of the FRLP Risk Score Model or GS Model and Chemotherapy Sensitivity

**FIGURE 9:**
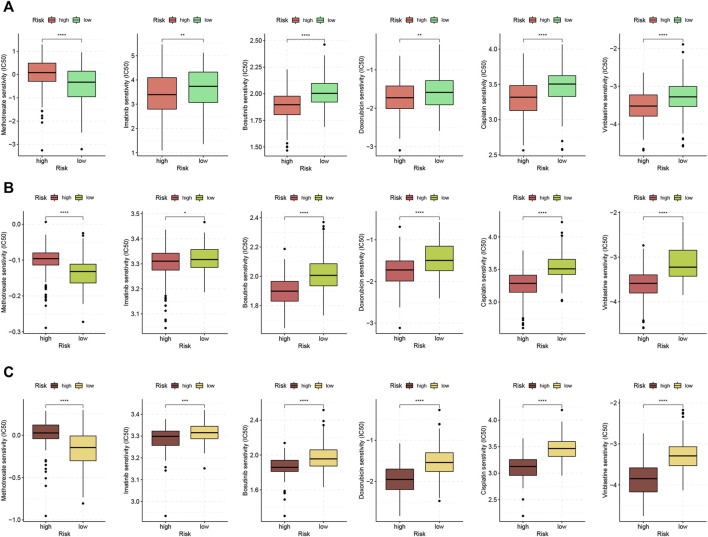
Chemotherapeutic response analysis. Boxplots comparing differences in half-inhibitory concentration (IC50) values of six anti-tumor drugs between high- and low-risk score groups in **(A)** TCGA, **(B)** GSE84437, and **(C)** GSE62254 (∗*p* < 0.05, ∗∗*p* < 0.01, ∗∗∗*p* < 0.001, ∗∗∗∗*p* < 0.0001).

The ssGSEA analysis revealed a higher enrichment level of carcinogenic pathways in the high-risk group. This indicated that high-risk patients may exhibit better responses toward chemotherapy. We estimated the IC50 of six anti-tumor drugs in samples from the TCGA cohort and two GEO cohorts. For TCGA patients, we compared the sensitivity between high- and low-risk groups of the FRLP risk score model. For GEO patients, we compared the sensitivity between high- and low-risk groups of the alternative GS model. As demonstrated in Figure 9, the sensitivity to imatinib, bosutinib, vinblastin, doxorubicin, and cisplatin was significantly higher in the high-risk patients than that of the low-risk patients. On the other hand, low-risk patients only displayed higher sensitivity to methotrexate.

### Construction of a Nomogram Based on GS Model

**FIGURE 10:**
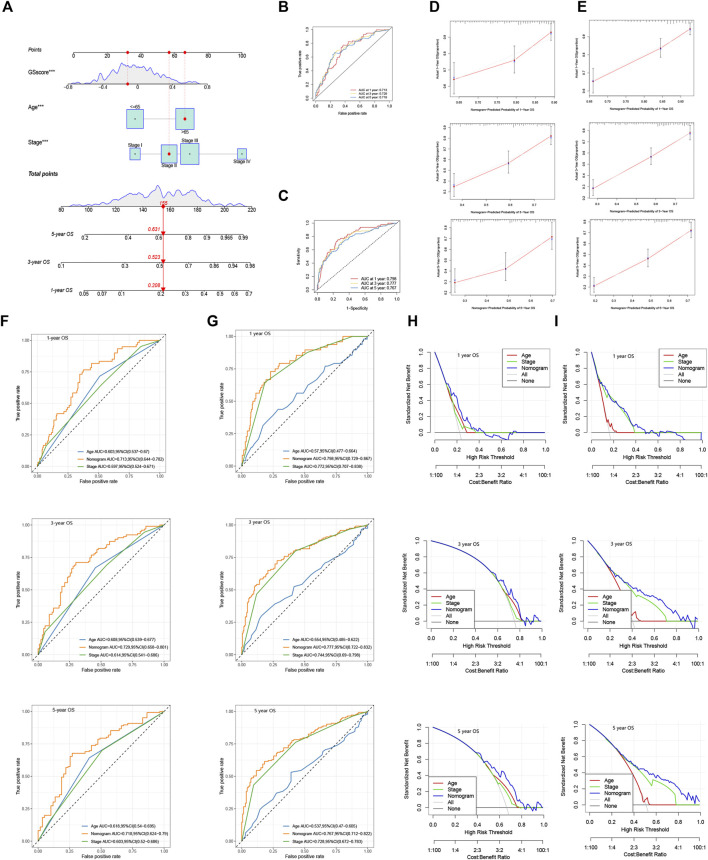
Construction of a nomogram using GS model. **(A)** Nomogram integrating GS score, age, and tumor stage for predicting 1, 3, and 5 year OS in TCGA and GSE62254. 1, 3, and 5 year ROC analyses of the nomogram in **(B)** TCGA and **(C)** GSE62254. Calibration curves of the nomogram for predicting 1, 3, and 5 year OS in **(D)** TCGA and **(E)** GSE62254. Time-dependent ROC curves evaluating the efficacy of the nomogram to predict 1, 3, and 5 year OS in **(F)** TCGA and **(G)** GSE62254. DCA curves estimating the predictive efficacy of the nomogram in **(H)** TCGA and **(I)** GSE62254.

Considering the inconvenience of using GS score directly in predicting patients' prognosis, we constructed a nomogram integrating GS score, age, and tumor stage based on TCGA cohort and GSE62254 (GSE84437 was excluded for lack of tumor stage profile) (Figure 10A). AUC analysis (Figures 10B,C) and calibration curves (Figures 10D,E) confirmed the high accuracy of the nomogram for predicting OS at 1, 3, and 5 years in both of the two cohorts. Furthermore, time-dependent ROC (Figures 10F,G) and DCA analysis (Figures 10H,I) was also performed in each of the cohort, which all confirmed the superior predicting ability of the nomogram compared with the age- or stage-only model.

### Development of Dynamic Nomograms Based on FRLP Risk Score Model and GS Model

At the end of the study, we generated two online dynamic nomograms based on FRLP risk and GS score model, respectively (https://ljzwhdx.shinyapps.io/FRLPdynanomo/; https://ljzwhdx.shinyapps.io/GSdynanomo/). Both of the two nomograms were in interactive forms, which could facilitate clinician’s prediction for prognosis. Supplementary Figure S5 displays the interface of the nomogram based on the FRLP risk score model. Predicted values of time-dependent survival probability could be easily obtained after selecting risk score or GS score, stage, and age of a specific GC patient.

## Discussion

Ferroptosis is a form of programmed cell death intimately associated with iron metabolism and peroxidation of polyunsaturated fatty acids (Stockwell et al., 2017). LncRNAs were able to regulate gene expression at both transcriptional and post-transcriptional levels (Statello et al., 2021). In recent years, the construction of prognostic prediction models based on ferroptosis-related lncRNAs has attracted particular attention of researchers (Table 1). However, these risk models concentrated on the specific expression of lncRNAs, which limited their clinical practicability. Two recently established risk score models independent of the exact lncRNA expressions caught our attention (Li et al., 2021; Tang et al., 2021). In the present study, we demonstrated that the similar risk score model of ferroptosis-related lncRNA pairs (FRLP) could also be applied to GC patients. The AUCs for the risk score to predict 1, 3, and 5 years’ OS were all over 0.70 according to the ROC curves. Aside from the high accuracy in predicting GC patients’ clinical outcomes, the FRLP risk score model was also closely connected with tumor microenvironment, biological function, and responses to chemo- and immunotherapies. High-risk patients of our FRLP risk score model were characterized by higher infiltration of immune cells (especially immunosuppressive and pro-tumorigenic cells), higher carcinogenic and stromal activities, and better sensitivity to some types of anti-tumor drugs. On the other hand, low-risk patients displayed better treatment responses to methotrexate and higher immunotherapy sensitivity. Considering that lncRNA expression profiles in the FRLP model were not available in microarray datasets, we also introduced an alternative gene set score (GS) model. We performed a series of analyses to confirm the high degree of compliance between FRLP and GS models. Subsequently, we validated the robustness of our FRLP risk score model in two external GEO cohorts using the alternative GS score, which confirmed the potential of applying the risk score model to a wider range of patients. Moreover, to enhance the clinical utility of the FRLP and the alternative GS risk score model, we also built two nomograms based on the two models, respectively. Finally, two dynamic nomograms with interactive interfaces based on the FRLP model and GS model were also constructed to further facilitate clinician’s prediction for prognosis.

The interaction between tumor cells and tumor infiltrating immunes (TIICs), namely the tumor microenvironment (TME), has been proved to play a crucial role in tumorigenesis. Some types of infiltrating immune cells have anti-tumor activities, such as CD8^+^ cytotoxic T lymphocytes (CTL), CD4^+^ T helper cells, natural killer cells, and dendritic cells. Conversely, regulatory T cells (Treg) and myeloid-derived suppressor cells (MDSCs) are regarded as “bad guys” in the TME (Pitt et al., 2016). Besides, some immune cells undergo polarization in the development of cancer and can exhibit either anti-tumor or tumor-promoting function depending on different cancer stages, such as neutrophils and macrophages (Ngambenjawong et al., 2017; Giese et al., 2019). Some works still regard these two kinds of cells tumor-promoting as their high infiltration level was frequently correlated with poor prognosis of many human tumor types (Mantovani et al., 2017; Shaul and Fridlender, 2019). Cancer-associated fibroblasts (CAFs) are another important kind of immunosuppressive cells that promote tumor progression by modulating angiogenesis and epithelial–mesenchymal transmission of cancer cells (Sahai et al., 2020).

Currently, the relationship between ferroptosis and immunity is quite ambiguous. Experimental evidence has already shown that interferon gamma (IFN-γ) released by CD8^+^ T cells promotes tumor cell ferroptosis (Wang et al., 2019). The negative association between CD8^+^ T cell infiltration, IFN-γ expression, and the expression of SLC3A2 and SLC7A11 was also shown in human melanoma tissues (Wang et al., 2019). A recent study revealed that CAFs exerted the tumor promoting role in gastric cancer by inhibiting ferroptosis in GC cells (Zhang et al., 2020). However, it is still immature to classify ferroptosis into the category of “immune-related cell death.” In the current study, we identified that the TME of high-risk group was characterized by higher stromal contents, as demonstrated by ESTIMATE and biological function analysis: high-risk patients exhibited higher stromal scores, and KEGG stromal-related pathways including “ECM–receptor interaction,” “focal adhesion,” “dilated cardiomyopathy,” “epithelial–mesenchymal transition,” “hypoxia,” and “angiogenesis” had a higher enrichment level in the high-risk group. As for the immune cells in TME, despite a positive correlation between the risk score and TIIC infiltration, there were no significant differences in the anti-tumor immune cell content between the two risk groups. On the other hand, pro-tumorigenic cells were more densely infiltrated in the TME of high-risk patients. It is also noteworthy that “GO” items related to cell migration and “Hallmark” items related to oncogenic activities were also enriched in the high-risk group. Thus, we speculated that the TME of the high-risk group was stroma-related and immunosuppressive, which may closely be linked with tumorigenesis.

Immune checkpoint is capable of inhibiting the over-activation of T cells and preventing autoimmune diseases. But under tumor circumstances, it will prevent T cells from approaching the tumor, weakening the ability of the immune system to recognize and destroy tumor cells (Tan et al., 2020). In recent years, immunotherapy targeting immune checkpoint modulation, namely the immune checkpoint blockage (ICB), has shown promising efficacy in cancer treatment (de Miguel and Calvo, 2020). However, the benefit, to date, has been limited to a minority of patients with certain cancer types (Sharma et al., 2017). TMB and TIDE scores are two effective methods to evaluate responses to ICB treatment. TMB is a quantitative measure of the total number of somatic non-synonymous mutations per coding area of a tumor genome (Melendez et al., 2018). Generally, high TMB suggests better OS in cancer patients after receiving ICB (Cao et al., 2019; Samstein et al., 2019; Valero et al., 2021). TIDE score was a computational method that combines two primary mechanisms in tumor evasion, T cell dysfunction and T cell exclusion. A higher tumor TIDE score is associated not only with worse ICB response but also with worse patient survival under anti-PD1 and anti-CTLA4 therapies (Jiang et al., 2018). In the present study, we observed a heightened expression of seven ICGs in the high-risk group. To figure out which group of GC patients displayed better ICB treatment response, we performed TMB and TIDE analyses in two groups of patients. Intriguingly, we found that high-risk STAD patients exhibited lower TMB and higher TIDE, which indicated that high-risk STAD patients may not actually benefit from ICB treatment though highly expressed seven ICGs. On the contrary, patients in low-risk groups may exhibit better immunotherapeutic response due to the relatively high TMB and low TIDE scores. IPS data collected from TCIA database also demonstrated that TCGA low-risk score or GEO low-GS patients were more likely to benefit from anti-CTLA4 and (or) anti-PD1 immunotherapy.

Finally, high-risk patients were demonstrated to exhibit higher sensitivity toward five kinds of chemotherapeutic or targeted drugs including vinblastin, cisplatin, doxorubicin, immatinib, and bosutinib. In contrast, low-risk patients solely responded to methotrexate. This perhaps could be attributed to two group’s discrepancy in ssGSEA analysis. The overactivity of carcinogenic pathways in high-risk patients, on the one hand, contributed to the shortened survival. On the other hand, however, this may also confer high-risk patients exhibiting better responses to a wider range of anti-tumor drugs. For instance, protein tyrosine kinase-associated pathways, such as IL6/JAK/STAT and PI3K/AKT, were highly enriched in high-risk patients, which may account for high-risk patients’ elevated sensitivity to tyrosine kinase inhibitors (TKIs) such as immatinib and bosutinib. Item “E2F target” was markedly enriched in the low-risk group according to ssGSEA analysis. Transcriptional factor E2F family plays a critical role in determining the time point of cell division. The expression of E2F targets gradually increases during G1 and must reach a threshold level in order for cells to pass the restriction point and progress to S phase. It was revealed that the activity of E2F peaks at G1-S transition gradually decreases during the phase and is totally repressed in both G2 and M phases (Kent and Leone, 2019). Therefore, the above analysis probably could explain why low-risk patients exhibited higher sensitivity to G1/S specific drug, methotrexate. Besides, the proportion of tumor cells arrested in the G2 phase was higher in low-risk patients due to higher enrichment level of “G2M checkpoint.” Hence, low-risk patients were less sensitive to the M phase-specific drug vinblastin. Our findings may help to optimize current chemotherapeutic strategy for GC patients.

There are several limitations of our present study. First, limited by the sequencing depth of GEO dataset, our external validation of the FRLP risk score signature used an alternative GS score, instead of the risk score, which may affect the robustness of the validation results. Second, all of our conclusions were drawn based on public databases; future large-scale and real-world studies are thus warranted.

## Conclusion

In this study, we developed an FRLP risk model composed of 10 differentially expressed ferroptosis lncRNA pairs that does not rely on the exact lncRNA expression level in the TCGA cohort. An alternative GS model, which shared a high degree of compliance with the FRLP model, was also constructed and used it to validate the application value of the FRLP model in two GEO microarray datasets. Two online dynamic nomograms with interactive interfaces were finally generated to facilitate clinician’s prediction for prognosis. The novel FRLP risk score signature established in the current study might provide insights for the accurate prediction and comprehensive management for GC patients.

## Data Availability

The datasets supporting the conclusions of this article are available in The Cancer Genome Atlas (https://portal.gdc.cancer.gov), Gene Expression Omnibus (https://www.ncbi.nlm.nih.gov/geo) and University of California Santa Cruz database (https://xena.ucsc.edu). The two online nomograms generated in the study could be accessed *via* https://ljzwhdx.shinyapps.io/FRLPdynanomo/ and https://ljzwhdx.shinyapps.io/GSdynanomo/.

## Supplementary Material

Supplementary Figure S1 Analytic process of the study.

Supplementary Figure S2 Violin plots exhibiting the infiltration differences of 10 immune cell types between high- and low-risk groups.

Supplementary Figure S3 Construction of the gene set score (GS) model, an alternative to the FRLP risk score model in TCGA cohort. **(A)** Volcano plot displaying genes upregulated and downregulated in high-risk patients, which were defined as gene sets A and B, respectively. **(B,C)** KEGG analysis of gene set A and gene set B. **(D)** Boxplots showing the differences in FRLP risk score, enrichment score of gene sets A and B, GS score in TCGA high- and low-FRLP risk groups. **(E)** Spearman correlation analysis of FRLP risk score and GS score. **(F)** Cut-off of the GS model. **(G)** Barplot and **(H)** Sankey diagram showing GS model’s representing ability of FRLP risk score model (*** *p*<0.001, **** *p*<0.0001).

Supplementary Figure S4 Boxplots showing differences in dysfunction score, exclusion score, and TIDE score of high- and low-GS groups in **(A)** GSE84437 and **(B)** GSE62254 (**** *p*<0.0001).

Supplementary Figure S5 The interface of online dynamic nomogram (https://ljzwhdx.shinyapps.io/FRLPdynanomo/) integrating FRLP risk score, tumor stage, and age for predicting time-independent survival probabilities in TCGA. **(A)** Input area for users to select stage (stages I–IV) or age (>65 or ≤65) as well as input the risk score and the follow-up time (futime). **(B)** Survival plots, showing patients' survival probabilities at different time points. **(C)** Predicted survival probabilities with a 95% confidence interval (CI), which could be obtained after inputting patient information in the input area. For example, when selecting “>65” for age, “stage III” for stage, and entering “3” for risk score, then a patient's 1-, 3-, and 5-year survival probabilities with a 95% CI are displayed using a black line, a blue line, and a red line, respectively. **(D)** Numerical summary showing the exact values of survival probabilities with a 95% CI.

Supplementary Table S1 Lists of genes in gene set A and gene set B.

## Abbreviations

AUC, area under the curve; DEL, differentially expressed lncRNA; DFS, disease-free survival; FRLP, ferroptosis-related lncRNA pair; GC, gastric cancer; GSEA, gene set enrichment analysis; GEO, Gene Expression Omnibus; GO, gene ontology; IC50, half-inhibitory concentration; IPS, immunophenoscore; ICG, immune checkpoint gene; ICB, immune checkpoint blockage; KEGG, Kyoto Encyclopedia of Genes and Genomes; LASSO, least absolute shrinkage and selection operator; OS, overall survival; ROC, receiver operating characteristic; ssGSEA, single sample gene set enrichment analysis; STAD, stomach adenocarcinoma; TCGA, the cancer genome database; TME, tumor microenvironment; TIIC, tumor infiltrating immune cell; TIDE, tumor immune dysfunction and exclusion; TCIA, The Cancer Immunome Atlas; UCSC, University of California Santa Cruz.
