# Corrigendum: A novel ferroptosis-related LncRNA pair prognostic signature predicts immune landscapes and treatment responses for gastric cancer patients

**DOI:** 10.3389/fgene.2022.1028480

**Published:** 2022-10-18

**Authors:** Jiazheng Li, Renshen Xiang, Wei Song, Jing Wu, Can Kong, Tao Fu

**Affiliations:** Department of Gastrointestinal Surgery II, Renmin Hospital of Wuhan University, Wuhan, China

**Keywords:** gastric cancer, ferroptosis, long non-coding RNA, prognostic model, tumor microenvironment, immunotherapy

In the original article, there were errors relating to the **Figures**, **Supplementary Material**, and within the article text (including typographical errors), as described below.

There were errors in some of the figures:

In Figure 2G, we used the 3-year ROC curve to get the best cut-off value because the AUC value of the risk score signature to predict 3-year OS was the highest according to Figure 2F. The corrected Figure 2 appears below.

**FIGURE 2:**
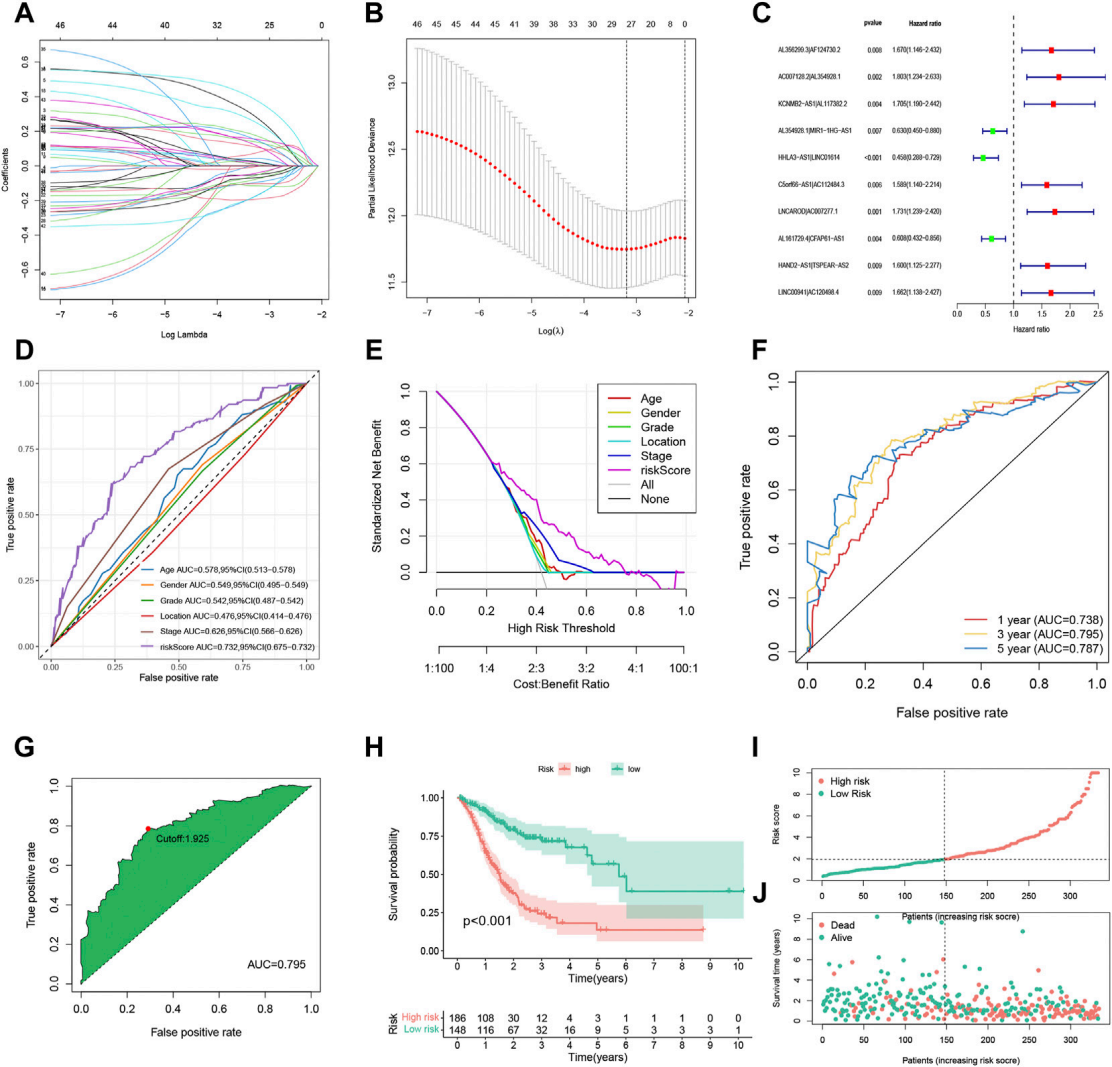
Construction and evaluation of the FRLP risk score model in TCGA cohort. **(A)** Least absolute shrinkage and selection operator (LASSO) coefficients of 27 prognosis-related FRLPs. **(B)** Tenfold cross-validation for tuning parameter selection in the LASSO model. **(C)** Forest plot showing 10 FRLPs identified by multivariate Cox regression analysis. **(D)** Receiver operating curve (ROC) comparing the risk score and other clinical factors in predicting total OS. **(E)** Decision curve analysis (DCA) curves estimating the predictive efficacy of the risk score from the perspective of clinical benefit. The *y*-axis refers to the net benefit. The *x*-axis refers to the predicted OS. The black line represents the hypothesis that all patients survive in 5 years. The gray line represents the hypothesis that no patients stay alive for more than 1 year. **(F)** ROC curve for predicting 1-, 3-, and 5-year overall survival (OS) of the FRLP risk score model. **(G)** Cut-off point of the risk score model. **(H)** Kaplan–Meier plot of high- and low-risk patients. **(I)** The risk score distribution. Green dots represent risk scores for low-risk patients; red dots represent risk scores for high-risk patients. **(J)** The relationship between survival status and risk score. The horizontal ordinate represents the number of patients; the vertical ordinate represents risk score (AUC, area under curve; CI, confidence interval).

In Figure 5, the TMB differences demonstrated by (A) were not clear**.** The corrected Figure 5 appears below.

**FIGURE 5:**
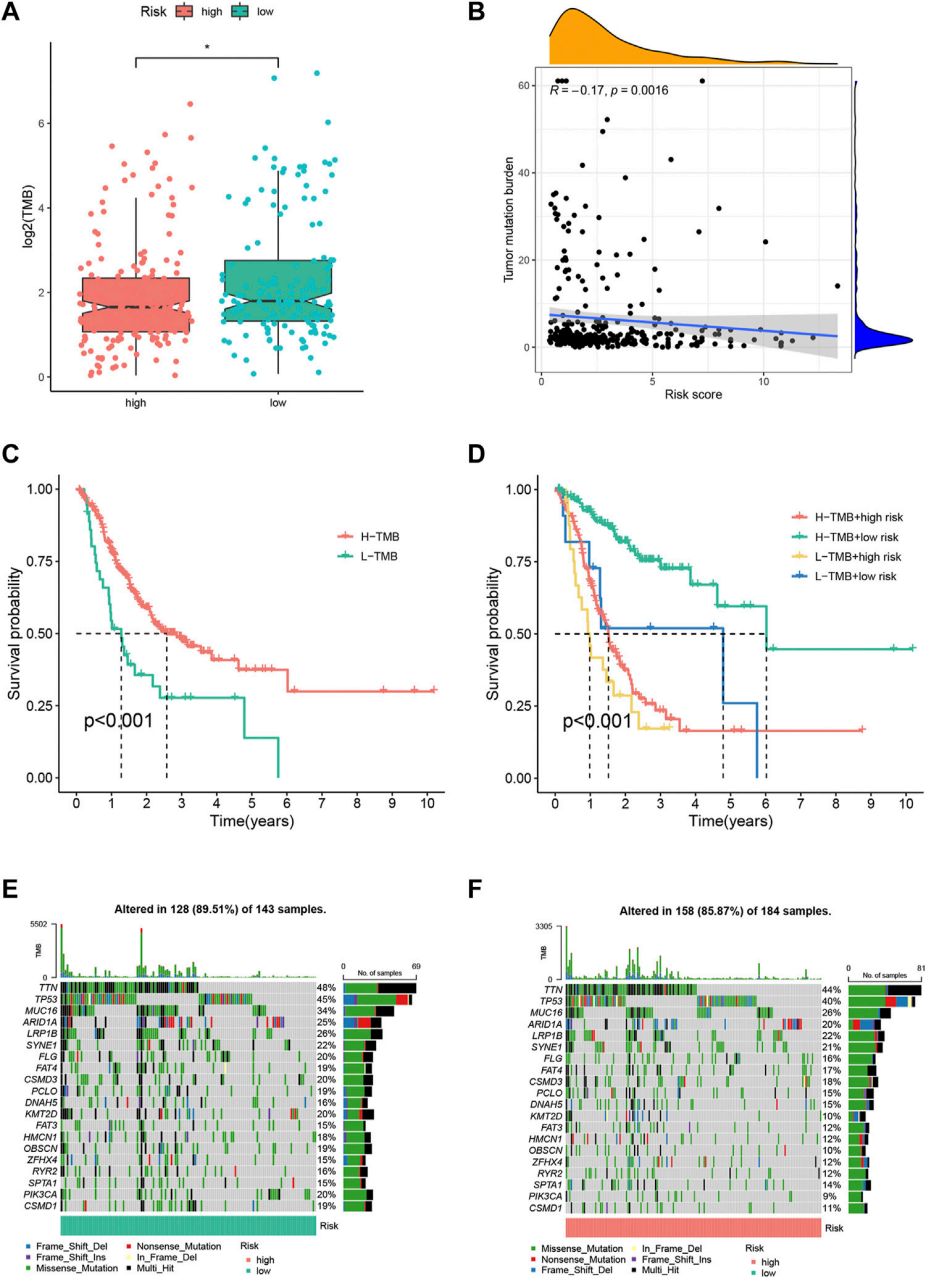
Tumor mutation burden (TMB) analysis of the FRLP risk score model. **(A)** TMB difference between high and low risk groups. **(B)** Correlation between the risk score and TMB. **(C)** Kaplan–Meier plots of patients with high and low TMB. **(D)** Kaplan–Meier curves of patients stratified by both TMB and the risk score. **(E,F)** Gene mutation analysis of patients in low and high-risk groups (*p < 0.05).

In Figure 6, the high and low risk groups in (D) were wrongly marked. The corrected Figure 6 appears below.

**FIGURE 6:**
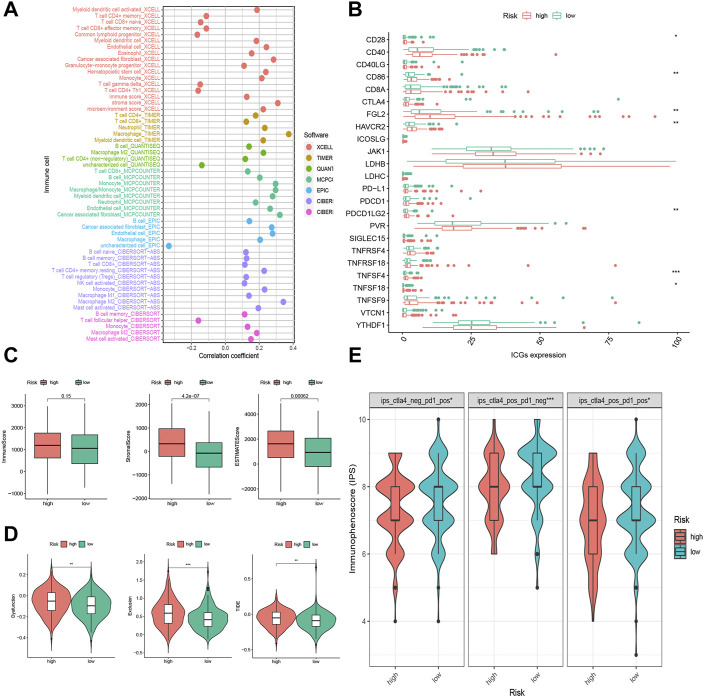
Tumor infiltrating immune cells (TIICs) and Immunotherapeutic sensitivity analysis of the FRLP risk score model. **(A)** The correlation between risk score and TIICs analyzed by seven different quantification methods of immune infiltration estimations including TIMER, xCell, quanTIseq, MCP-counter, EPIC, CIBERSORT-ABS, and CIBERSORT. **(B)** Expression of 24 immune checkpoint genes in high- and low-risk groups. **(C)** Boxplots showing Immune score, Stromal score, and ESTIMATE score in high- and low-risk groups. **(D)** Boxplots showing Dysfunction score, Exclusion score, and tumor immune dysfunction and exclusion (TIDE) score differences between high- and low-risk score groups. **(E)** Immunophenoscore (IPS) differences for ICB treatment between high- and low-risk groups; ips_ctla4_neg_pd1_pos refers to CTLA4-negative response and PD1-positive response; ips_ctla4_pos_pd1_neg refers to CTLA4-positive response and PD1-negative response; ips_ctla4_pos_pd1_pos refers to CTLA4-positive response and PD1-positive response (*p < 0.05, **p < 0.01, ***p < 0.001).

There were errors in the legends for Figures 6B,C and Supplementary Figure S5. In Figures 6B,C the legends were swapped and in Supplementary Figure S5, the URL was incorrect. The corrected legends appear below:

“Figure 6(B): Expression of 24 immune checkpoint genes in high- and low-risk groups.

Figure 6(C): Boxplots showing immune score, stromal score, and ESTIMATE score in high- and low-risk groups.

Supplementary Figure 5: The interface of online dynamic nomogram (https://ljzwhdx.shinyapps.io/FRLPdynanomo/) integrating FRLP risk score, tumor stage, and age for predicting time-independent survival probabilities in TCGA. **(A)** Input area for users to select stage (stages I–IV) or age (>65 or ≤65) as well as input the risk score and the follow-up time (futime). **(B)** Survival plots, showing patients’ survival probabilities at different time points. **(C)** Predicted survival probabilities with a 95% confidence interval (CI), which could be obtained after inputting patient information in the input area. For example, when selecting “>65” for age, “stage III” for stage, and entering “3” for risk score, then a patient’s 1-, 3-, and 5-year survival probabilities with a 95% CI are displayed using a black line, a blue line, and a red line, respectively. **(D)** Numerical summary showing the exact values of survival probabilities with a 95% CI.”

There were also errors in the in-text citation of Figures 6A–C, described below:

A correction has been made to the **Results**, subsection: “The Correlation of the FRLP Risk Score Model and Tumor Microenvironment,” Paragraph 1. The sentence previously stated: “The results demonstrated that the stromal score and ESTIMATE score were higher in the high-risk group, while there was no significant difference in the immune score between the two groups (Figure 6A). High-risk scores were more closely linked with high TIICs (Figure 6B).” The corrected sentence appears below:

“The results demonstrated that the stromal score and ESTIMATE score were higher in the high-risk group, while there was no significant difference in the immune score between the two groups (Figure 6C). High-risk scores were more closely linked with high TIICs (Figure 6A).”

A correction has also been made to the **Results**, sub-section: “The Correlation of the FRLP Risk Score Model and Immunotherapeutic Sensitivity,” Paragraph 1.

The sentence previously stated: “Of all the 24 selected ICGs, CD28, CD86, FGL2, HAVCR2, PDCD1LG2, TNFSF4, and TNFSF18 were highly expressed in the high-risk group (Figure 6C).” The corrected sentence appears below:

“Of all the 24 selected ICGs, CD28, CD86, FGL2, HAVCR2, PDCD1LG2, TNFSF4, and TNFSF18 were highly expressed in the high-risk group (Figure 6B).”

There was an error in the original article text, as there were seven differentially expressed ICGs instead of six. A correction has been made to the **Discussion,** Paragraph 4.

The sentence previously stated: “In the present study, we observed a heightened expression of six ICGs in the high-risk group […], which indicated that high-risk STAD patients may not actually benefit from ICB treatment though highly expressed six ICGs.” The corrected sentence appears below:

“In the present study, we observed a heightened expression of seven ICGs in the high-risk group […], which indicated that high-risk STAD patients may not actually benefit from ICB treatment though highly expressed seven ICGs.”

Finally, some minor typographical errors in the article text have been corrected.

The comma was placed at the wrong position.

A correction has been made to **Materials and Methods**, “Risk Score Model Construction,” Paragraph 1. This sentence previously stated: “Using R package “glmnet,”.” The corrected sentence appears below:

“Using R package “glmnet”,”.

In the original published article, there was an error in the main text: an excessive dash was wrongly typed.

A correction has been made to **Materials and Methods**, “Tumor Microenvironment Analysis,” Paragraph. This sentence previously stated: “TIMER, xCell, quanTIseq, MCP-counter, EPIC, CIBERSORT-ABS, and -CIBERSORT.” The corrected sentence appears below:

“TIMER, xCell, quanTIseq, MCP-counter, EPIC, CIBERSORT-ABS, and CIBERSORT.”

In the original article, there was an error in the main text, and the period was placed in the wrong position.

A correction has been made to **Results**, “The Correlation of the FRLP Risk Score Model and Tumor Microenvironment,” Paragraph 1. This sentence previously stated: “and ESTIMATE scores of two risk groups by the R package “ESTIMATE.”.” The corrected sentence appears below:

“and ESTIMATE scores of two risk groups by the R package “ESTIMATE”.”.

In the original article, there was an error in the main text and a letter “s” when typing the word “patients.”

A correction has been made to **Results**, “Construction of a Nomogram Based on GS Model,” Paragraph 1. This sentence previously stated: “Considering the inconvenience of using GS score directly in predicting patient’ prognosis,”. The corrected sentence appears below:

“Considering the inconvenience of using GS score directly in predicting patients’ prognosis,”.

The authors apologize for these errors and state that they do not change the scientific conclusions of the article in any way. The original article has been updated.

