# Supplementary figures and images for: A Novel Ferroptosis-Related LncRNA Pair Prognostic Signature Predicts Immune Landscapes and Treatment Responses for Gastric Cancer Patients
Source: Front Genet. 2022 Jun 20;13:899419. doi: 10.3389/fgene.2022.899419 (PMC9250987; doi:10.3389/fgene.2022.899419)

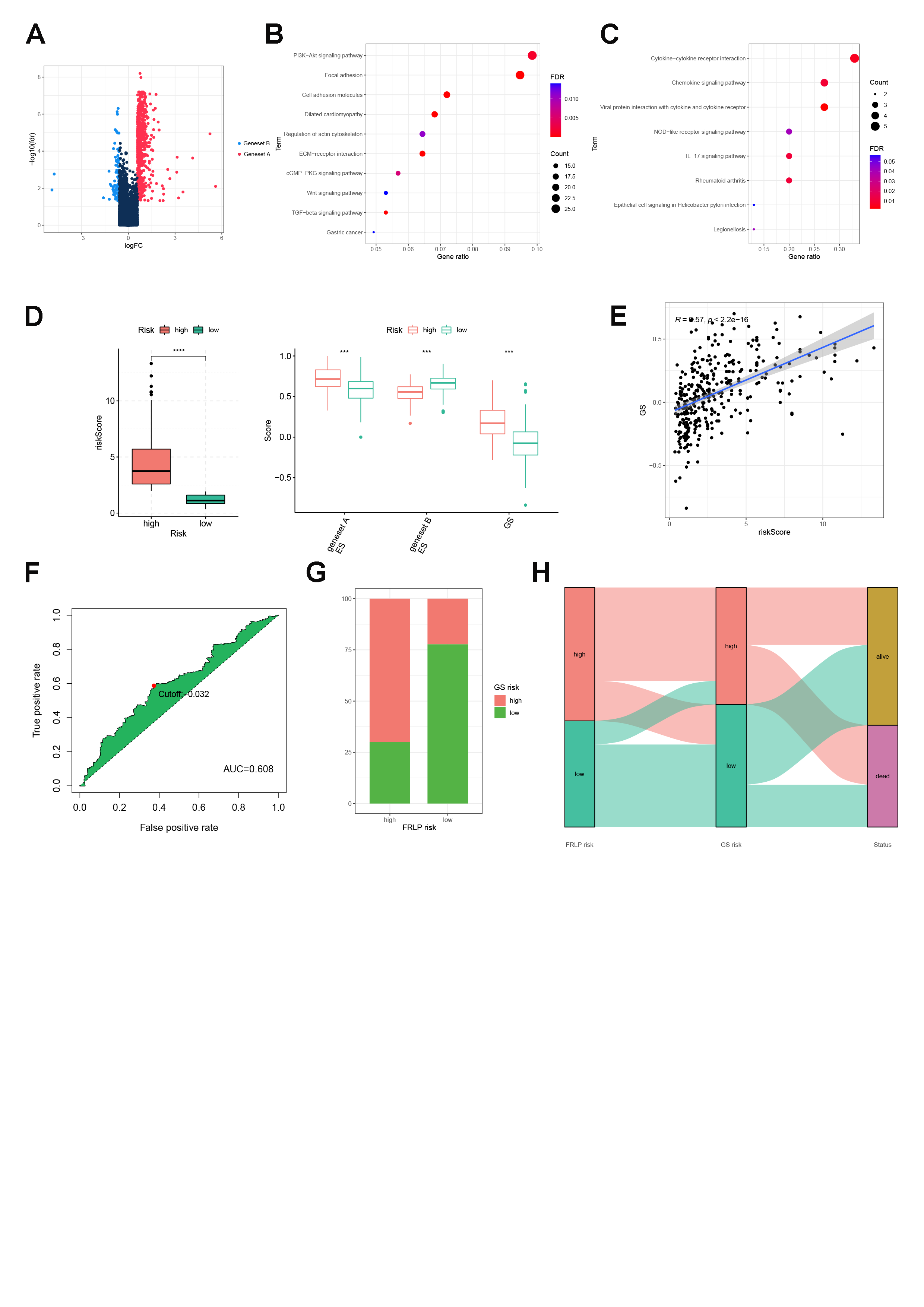

Supplement: Supplementary file 1 [file Image3.TIF]

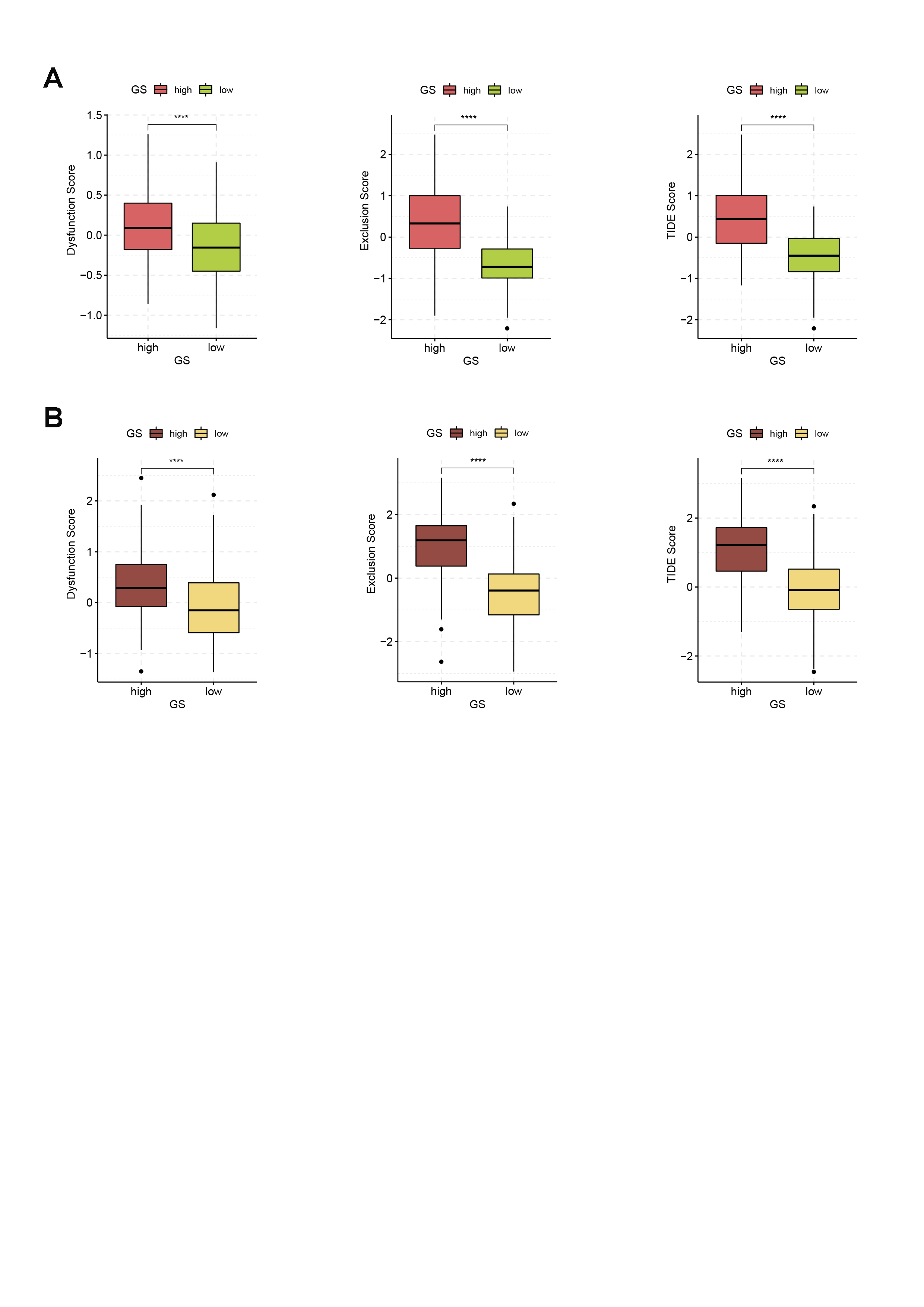

Supplement: Supplementary file 2 [file Image4.TIF]

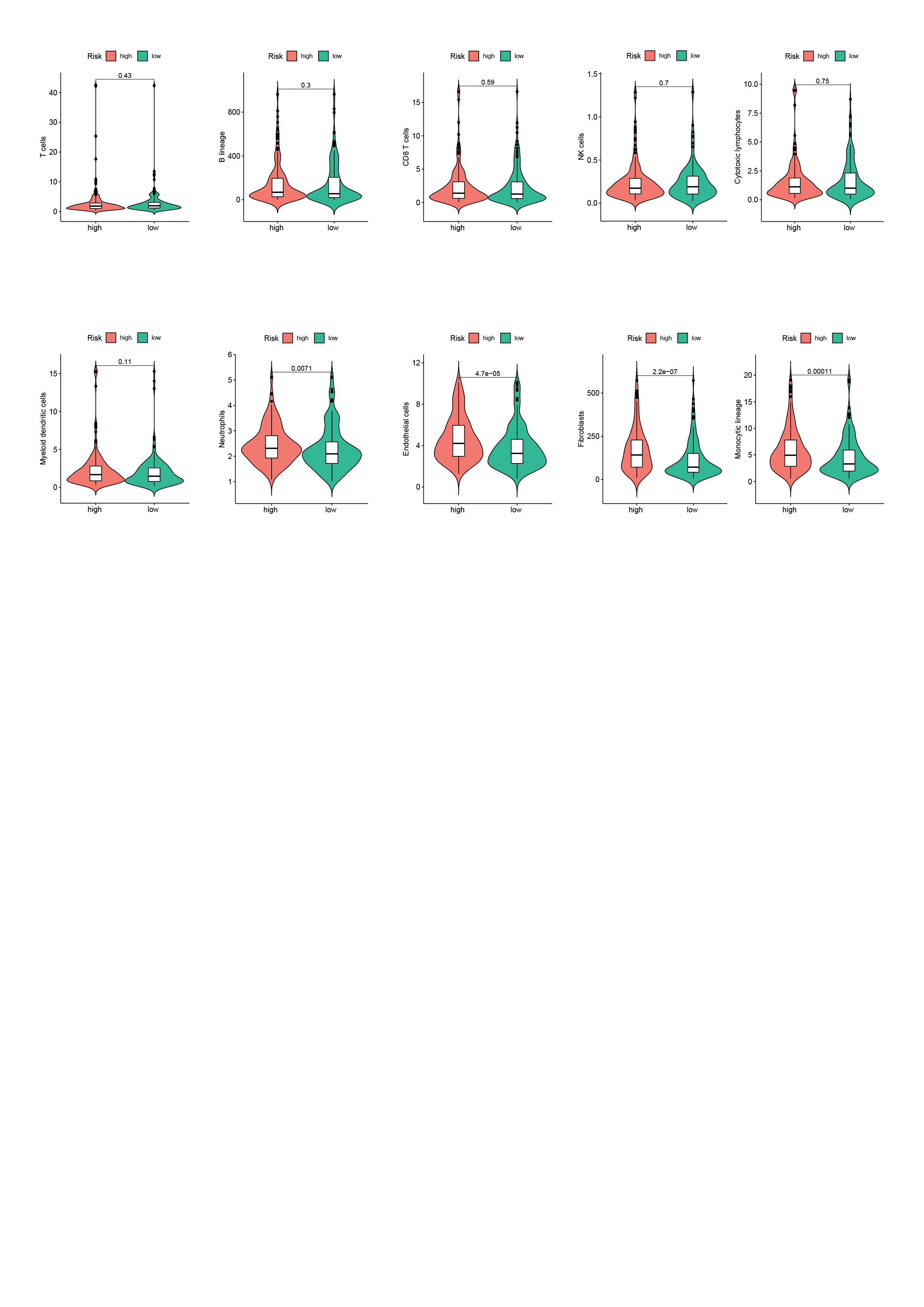

Supplement: Supplementary file 3 [file Image2.TIF]

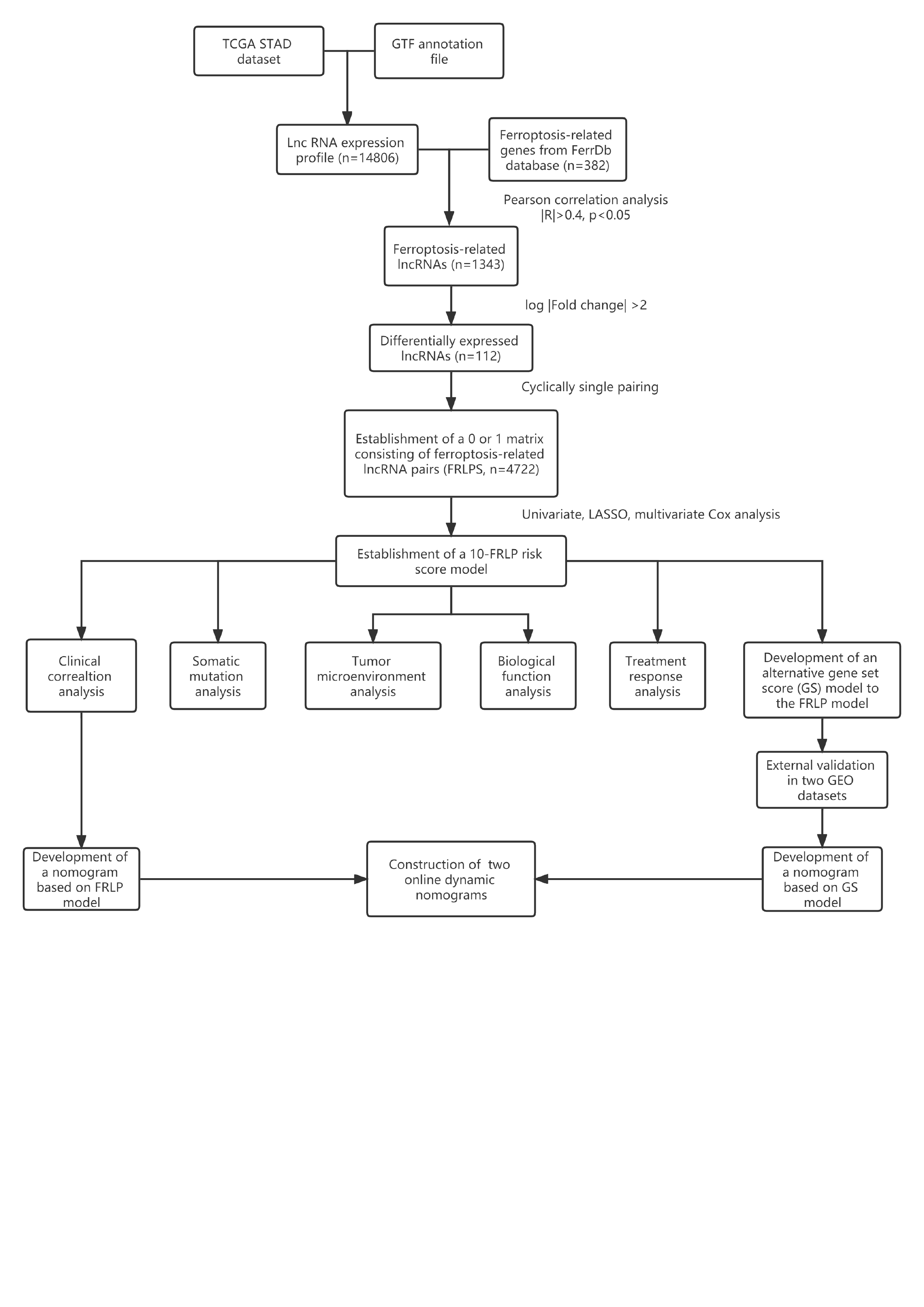

Supplement: Supplementary file 4 [file Image1.TIF]

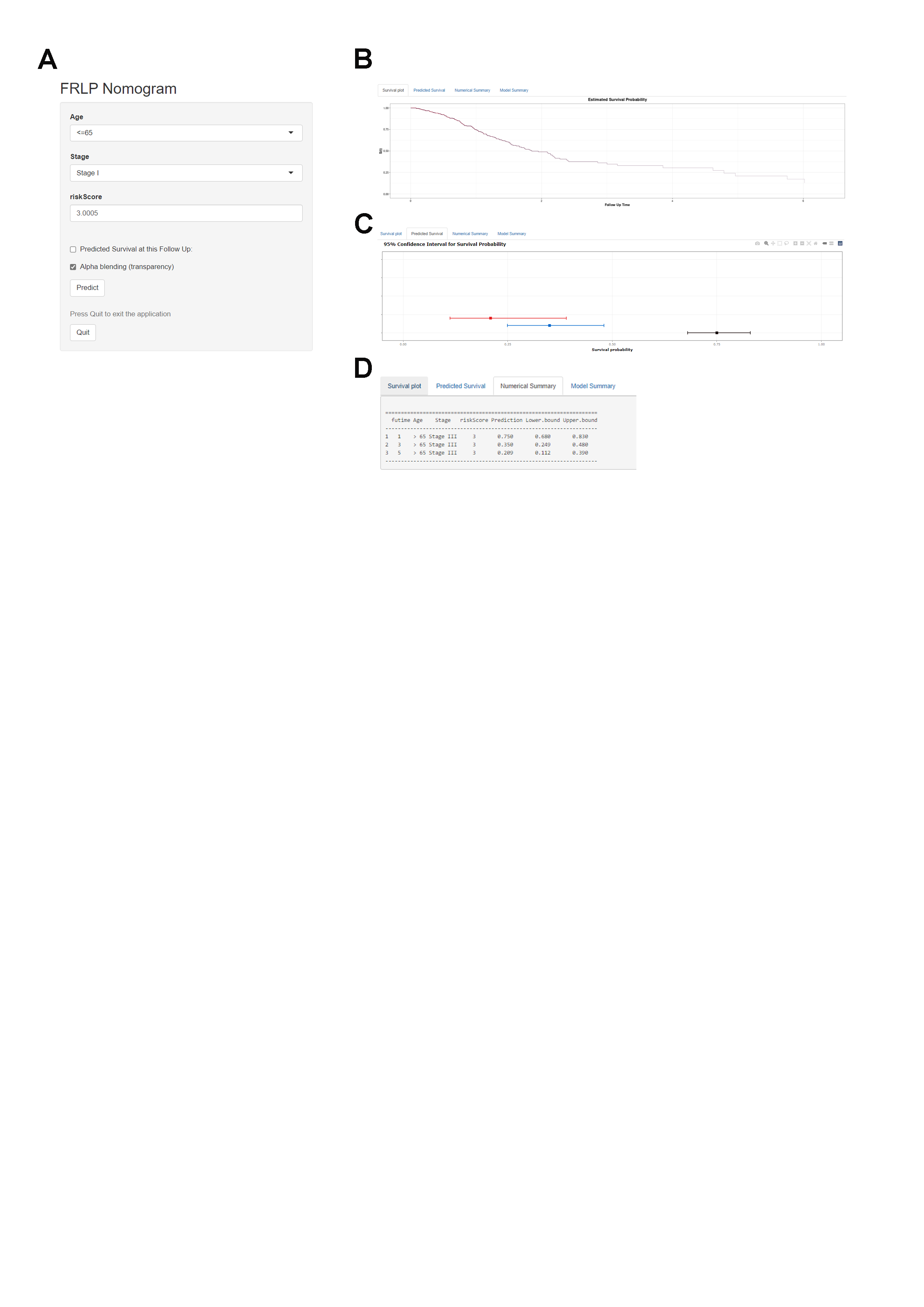

Supplement: Supplementary file 6 [file Image5.TIF]
